# An Unusual New Theropod with a Didactyl Manus from the Upper Cretaceous of Patagonia, Argentina

**DOI:** 10.1371/journal.pone.0157793

**Published:** 2016-07-13

**Authors:** Sebastián Apesteguía, Nathan D. Smith, Rubén Juárez Valieri, Peter J. Makovicky

**Affiliations:** 1 Área de Paleontología. Fundación de Historia Natural 'Félix de Azara', CEBBAD, Univ. Maimónides, Buenos Aires, Argentina; 2 Dinosaur Institute, Natural History Museum of Los Angeles County, Los Angeles, United States of America; 3 Secretaría de Cultura, Gobierno de la Provincia de Río Negro, General Roca, Argentina; 4 Section of Earth Sciences, Integrative Research Center, Field Museum of Natural History, Chicago, Illinois, United States of America; Raymond M. Alf Museum of Paleontology, UNITED STATES

## Abstract

**Background:**

Late Cretaceous terrestrial strata of the Neuquén Basin, northern Patagonia, Argentina have yielded a rich fauna of dinosaurs and other vertebrates. The diversity of saurischian dinosaurs is particularly high, especially in the late Cenomanian-early Turonian Huincul Formation, which has yielded specimens of rebacchisaurid and titanosaurian sauropods, and abelisaurid and carcharodontosaurid theropods. Continued sampling is adding to the known vertebrate diversity of this unit.

**Methodology/ Principal Findings:**

A new, partially articulated mid-sized theropod was found in rocks from the Huincul Formation. It exhibits a unique combination of traits that distinguish it from other known theropods justifying erection of a new taxon, *Gualicho shinyae* gen. et sp. nov. *Gualicho* possesses a didactyl manus with the third digit reduced to a metacarpal splint reminiscent of tyrannosaurids, but both phylogenetic and multivariate analyses indicate that didactyly is convergent in these groups. Derived characters of the scapula, femur, and fibula supports the new theropod as the sister taxon of the nearly coeval African theropod *Deltadromeus* and as a neovenatorid carcharodontosaurian. A number of these features are independently present in ceratosaurs, and *Gualicho* exhibits an unusual mosaic of ceratosaurian and tetanuran synapomorphies distributed throughout the skeleton.

**Conclusions/ Significance:**

*Gualicho shinyae* gen. et sp. nov. increases the known theropod diversity of the Huincul Formation and also represents the first likely neovenatorid from this unit. It is the most basal tetatanuran to exhibit common patterns of digit III reduction that evolved independently in a number of other tetanuran lineages. A close relationship with *Deltadromaeus* from the Kem Kem beds of Niger adds to the already considerable biogeographic similarity between the Huincul Formation and coeval rock units in North Africa.

## Introduction

The fossiliferous Huincul Formation (Late Cenomanian-Early Turonian) is widely exposed in Neuquén and Río Negro provinces of northern Patagonia, Argentina. It has yielded a diverse dinosaur fauna, including the remains of some of the largest dinosaurs known such as the titanosaurian sauropod *Argentinosaurus* [1], and the giant carcharodontosaurid theropod *Mapusaurus* [2]. Other notable dinosaur finds from this unit are the rebbachisaurid sauropod *Cathartesaura* [3], and the mid-sized abelisauroids *Ilokelesia* [4] and *Skorpiovenator* [5].

The senior author (S.A.) discovered a fossiliferous stretch of continuous Huincul Formation outcrops on the Río Negro shore of the Ezequiel Ramos Mexía Reservoir, in 1999. A joint expedition in 2007 that included three of the authors explored this set of exposures informally dubbed "Violante's field," and made several discoveries, including parts of a sauropod skeleton, a maxilla and several vertebrae of an abelisaurid theropod, and numerous dinosaur coprolites [6]. The most significant find, however, was an articulated partial skeleton of an unusual, mid-sized theropod dinosaur comprising a short section of the dorsal vertebral column, a distal section of the tail, the left scapulocoracoid and forelimb, and parts of both hind limbs. This specimen was subsequently excavated and prepared by staff of the Museo Patagónico de Ciencias Naturales.

The new specimen exhibits a new and unusual combination of derived characters that warrant recognition of a new taxon. Chief among these is a didactyl manus reminiscent of Late Cretaceous Laurasian tyrannosaurids, but the new specimen also exhibits derived traits otherwise only found in ceratosaurian theropods, as well as basal tetanurans. Here we describe the new theropod taxon and discuss its affinities and biogeographic implications, as well as examining forelimb reduction among theropods in light of this new discovery.

## Materials and Methods

### Material

The specimen described here was discovered on a paleontological expedition led by the senior author with the participation of NDS and PJM (see S1 Fig). A letter granting permission to prospect and collect fossils was issued to the senior author by Marcelo Solorza, who was Director of the Agencia Cultura de la Provincia de Río Negro at the time. As per agreement in this letter, all materials collected during the expedition were deposited in the Museo Patagónico de Ciencias Naturales, a provincially supported public repository in General Roca, Provincia de Río Negro. The new theropod specimen is housed and cataloged at that institution as MPCN PV 0001.

### Nomenclatural Acts

The electronic edition of this article conforms to the requirements of the amended International Code of Zoological Nomenclature, and hence the new names contained herein are available under that Code from the electronic edition of this article. This published work and the nomenclatural acts it contains have been registered in ZooBank, the online registration system for the ICZN. The ZooBank LSIDs (Life Science Identifiers) can be resolved and the associated information viewed through any standard web browser by appending the LSID to the prefix “http://zoobank.org/”. The LSID for this publication is: urn:lsid:zoobank.org:pub: B0A88805-53FE-4499-9B0E-E51E60AD2A6D. The electronic edition of this work was published in a journal with an ISSN, and has been archived and is available from the following digital repositories: PubMed Central, LOCKSS.

### Institutional abbreviations

In the description below we refer to specimens housed in the following publicly accessible repositories: **CCG**, Museum of Chendgu, College of Geology, Chengdu, China; **FMNH**, Field Museum of Natural History, Chicago, Illinois, USA; **MACN**, Museo Argentino de Ciencias Naturales 'Bernardino Rivadavia', Buenos Aires; **MB**, Humboldt Museum für Naturkunde, Berlin, Germany; **MCNA**, Museo de Ciencias Naturales y Antropológicas (J. C. Moyano) de Mendoza, Mendoza Province, Argentina; **MPCN PV**, Museo Patagónico de Ciencias Naturales, General Roca, Río Negro Province, Argentina; **MUCP**, Museo de la Universidad Nacional del Comahue, Neuquén Province, Argentina; **NHMUK** (formerly BMNH), Natural History Museum, London, United Kingdon; **RTMP**, Royal Tyrell Museum of Paleontology, Drumheller, Canada; **SGM**, Ministére de l'Énergie et des Mines, Rabat, Morocco; **UCMP**, University of California Museum of Paleontology, Berkeley, California, USA; **UMNH**, Natural History Museum of Utah, Salt Lake City, Utah, USA; **YPM**, Peabody Museum of Natural History, Yale University, New Haven, Connecticut, USA. For clarity, we cite specimen numbers when making comparisons to direct observations on fossil specimens or our photographs thereof, and cite literature when observations are taken from other authors.

### Phylogenetic analysis

The skeleton of *Gualicho* exhibits an unusual combination of character states. Some, such as the didactyl manus with a semilunate distal carpal are indicative of derived tetanuran affinities [7], whereas others, such as the expanded posterior margin of the metatarsal III proximal articulation, are shared with ceratosaurs [8]. In order to infer the relationships of this unusual species, we added it to a recently published, broadly sampled analysis of basal tetanuran phylogeny that also includes a number of ceratosaurian taxa [7]. We made edits in character coding (see S1 Text), which did not alter the strict consensus tree topology presented by [7], and also added nine new characters. The modified matrix is available as S1 File. Taxon sampling was increased by including not only *Gualicho*, but also the roughly coeval *Deltadromeus* [9], as the two taxa share some unique anatomical traits in the pectoral girdle. Both taxa are quite fragmentary, and the proportion of missing data in *Gualicho* (76.3%) and *Deltadromeus* (78.8%) exceed the median value (63.8%) for the entire data set.

The matrix was analyzed with Maximum Parsimony in the freeware TNT [10]. One thousand random addition replicates were subjected to the Tree Bisection and Rearrangement (TBR) algorithm, with 10 trees retained for each replicate. The stored shortest trees recovered were then subjected to an additional round of TBR branch swapping to obtain all shortest trees. The data were also analyzed using the "new technologies" option, to ensure that the shortest trees had been obtained. The shortest trees obtained were subjected to strict consensus and reduced consensus techniques to summarize common branching patterns.

Based on the results of the first analysis, which recovered *Gualicho* as sister to neovenatorid allosauroids including megaraptorans, we also added *Gualicho* as a terminal taxon to a recently published dataset [11], which is slightly modified from a previous study [12], and which posits tyrannosauroids as closely related to megaraptorans. The aim of this was to test the possibility whether the didactyl manus of *Gualicho* could be a synapomoprhy shared with Tyrannosauridae. As with the Carrano et al. [7] matrix, we edited scorings and added characters (See S1 Text), but in this case editing led to different most-parsimonious topologies from those originally published by the authors. The modified matrix is available as S2 File. Addition of *Gualicho* did not alter the branching pattern between taxa in the new topologies, when subjected to parsimony analysis using the same protocol as outlined above, suggesting that character re-scoring and unordering of characters rather than taxon addition led to the observed topological changes. The original authors ordered many multistate characters (~10% of all characters were ordered), but we reran the modified dataset with all characters unordered in keeping with the modified Carrano et al. [7] dataset and to determine what effect this parameter had on results.

### Multivariate analyses

The unusual forelimb of *Gualicho* invites comparisons to other clades with reduced forelimbs. Beyond examining the topologies and character state optimizations resulting from the cladistic analysis, we addressed the question of whether *Gualicho* shares aspects of forelimb reduction with other clades, or whether these evolved in parallel, through multivariate analyses. We examined forelimb disparity in theropods in two complementary ways. Firstly, we followed an approach originally advocated by Foote [13] and employed recently in vertebrate paleobiology by several authors [14–16] by conducting a Principal Coordinates (PCO) analysis on the forelimb character data from the main phylogenetic dataset [7]. We used all 33 characters related to the humerus, forearm, carpus, and manus, (S2 Text) employing the Gower metric to derive a morphospace so as to downweight the influence of multistate characters over binary ones, and also because this metric accommodates missing data [13]. Missing data forced us to reduce the dataset to 34 of the original taxa from the phylogenetic dataset that permit scoring of more than 46% of the traits. We also added six taxa (*Eoabelisaurus*, *Guanlong*, *Albertosaurus*, *Harpymimus*, *Ornithomimus*, *Tanycolagreus*) which exhibit some degree of forelimb reduction in a broad sense (i.e. reduction in muscle attachments or articulations as well as elemental shortening), but which were not part of the Carrano et al. [7] matrix. The PCO matrix is available as S1 Table.

Our second approach was to conduct a morphometric analysis. We added length measurements of the humerus, ulna, metacarpal I, and femur of MPCN PV 0001 to a published dataset of theropod limb measurements [17]. The data set was sorted, so that only taxa with complete forelimb (humeral, radial and MC I lengths) and femoral measurements were included and logarithmically transformed. Avian taxa including scansoriopterygids were also excluded to amplify differences between non-avian theropod taxa, and because they are phylogenetically remote from *Gualicho*. The final data set included 61 taxa (S2 Table). Data were subjected to phylogenetic Principal Components Analysis (pPCA) [18] in the R software environment [19]. A phylogenetic tree for the 61 taxa was constructed to reflect the results of the first phylogenetic analysis above based on the Carrano et al. [7] data set. Numerous taxa in the pPCA analysis were not included in our phylogenetic analysis, so their relationships were resolved to follow recent analyses that infer relationships within those clades. Relationships within Ceratosauria follow [20], and relationships among Coelurosauria are taken from [21]. Branch lengths were calibrated stratigraphically and zero-branch lengths were corrected using the EQUAL method using the STRAP package [22] in R [19]. A Nexus-formatted file with the tree is available as S3 File, a text file with taxon ages for calibrating the tree is available as S4 File, a data file of log-transformed measurements formatted for R analysis is provided in S3 Table and a text file with the R script to execute the pPCA is available in S5 File. Following analysis of the data, we exported pPCA scores from R to PAST [23] for graphical manipulation, and convex hulls were plotted for individual clades to facilitate comparisons. As noted by Benson and Choiniere [17], the first phylogenetic Principal Component (PC) axis is largely driven by size, as revealed by the strong (close to -1) and similar loadings on all four parameters, so comparisons focused on phylogenetic Principal Components 2 and 3. Forelimb data were analyzed with femoral length included as a proxy for body size. We ran separate analyses implementing either a Brownian Motion model or a Lambda model for the pPCA analysis and used the covariance method to calculate Principal Component scores, loadings and other variables. Recent studies have noted that model choice can bias pPCA results [24], but suitable methods to address this concern are still under development, hence our choice to implement both available models. We also performed a traditional Principal Components Analysis in PAST [23] to contrast with the pPCA.

## Results

### Systematic paleontology

Dinosauria

Theropoda

Tetanurae

Avetheropoda

*Gualicho shinyae* gen. et sp. nov. (replaces *Nototyrannus violantei* Anonymous, 2011, nomen nudum)

urn:lsid:zoobank.org:act:A793B82A-972E-4159-9D88-CCEB9CBDECB5

#### Holotype

**Fig 1:**
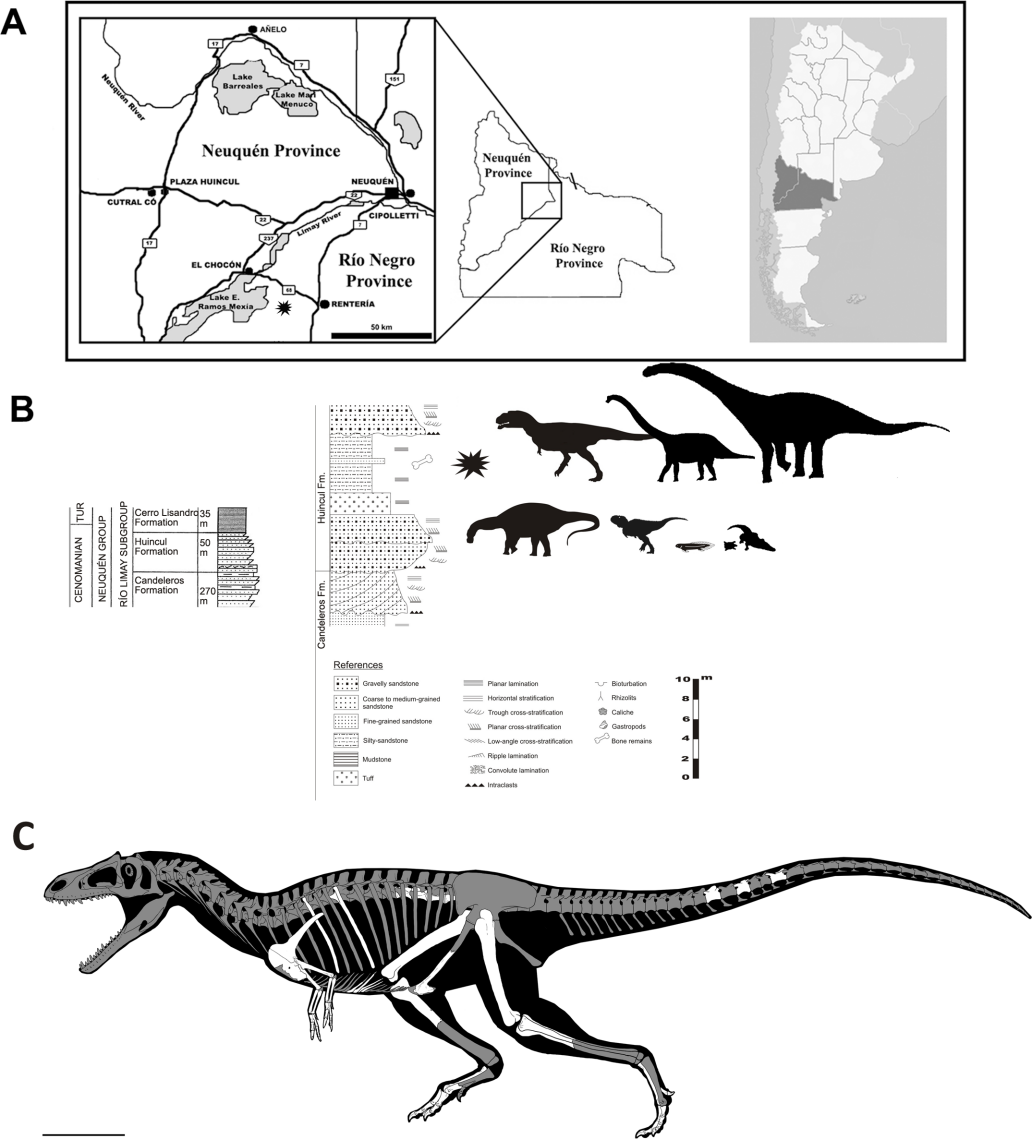
Life reconstruction of skeletal remains of *Gualicho shinyae* and stratigraphic and geographic details of the find. (A) Map of Rio Límay region of northern Patagonia, showing where the holotype of *Gualicho shinyae* was discovered (star) (B) Schematic stratigraphic column of lower part of Neuquén Group (Upper Cretaceous) strata exposed in the Neuquén Basin with approximate level at which the holotype of *Gualicho shinyae* was collected from the base of the Huincul Formation. See S1 Fig for excavation photos. (C) Skeletal reconstruction of *Gualicho shinyae* showing recovered elements in white and missing elements in grey shading. Artwork by J. González.

MPCN PV 0001, comprising four articulated centra from the dorsal vertebral column, an articulated gastral basket, a section of the tail distal to the transition point, the left scapulocoracoid and forelimb, the distal end of both pubes including the pubic boot, and parts of both hind limbs (Fig 1A). Much of the specimen had been lost to erosion when discovered, but the preserved parts including the forelimb, dorsal vertebrae, gastralia, and feet were articulated. Specimen measurements are provided in Table 1.

**Table 1:** Select measurements of the holotype specimen of *Gualicho shinyae*.

Element	Dimension measured	Measurement (mm)
**Dorsal (1st in series)**	centrum length	92
**Dorsal (2nd in series)**	centrum length	104
**Dorsal (3rd in series)**	centrum length	80†
**Caudal (1st in series)**	centrum length	72
**Caudal (2nd in series)**	centrum length	83
**Caudal 3rd in series)**	centrum length	50*
**Scapula**	Total length	439†
	Blade dorsoventral width (minimum)	33
	Blade dorsoventral width (at base)	75
**Coracoid**	Total dorsoventral length	168†
**Humerus**	Length	286
	Deltopectoral crest to internal tuberosity	96
	Proximal mediolateral width	78.07
	Proximal anteroposterior width	20.99
	Midshaft mediolateral width	25.92
	Midshaft anteroposterior width	26.18
	Midshaft circumference	78.66
	Deltopectoral crest length	35.60*
	Estimated deltopectoral crest length	56.5
	Deltopectoral crest width	21.42
	Distal mediolateral width	43.74
	Distal anteroposterior width	27.26
**Radius (right)**	Length	155.4
	Proximal mediolateral width	20.8
	Proximal anteroposterior width	14.11
	Distal mediolateral width	17.92
	Distal anteroposterior width	18.22
**Radius (left)**	Length	152.75
	Proximal mediolateral width	19.76
	Proximal anteroposterior width	15.24
	Distal mediolateral width	14.57
	Distal anteroposterior width	22.18
**Ulna (right)**	Length	166.8
	Proximal mediolateral width	14.27*
	Proximal anteroposterior width	26.36
	Olecranon height above articulation	11.25
	Distal mediolateral width	19.75
	Distal anteroposterior width	13.03
**Ulna (left)**	Length	157.66*
	Proximal anteroposterior width	22.8*
	Distal mediolateral width	20.72
	Distal anteroposterior width	12.29
**Scapholunare (left)**	Mediolateral width	11.91
	Anteroposterior width	15.43
**Semilunate carpal (left)**	Mediolateral width	17.13
	Anteroposterior width	18.48
**MC I (left)**	Length	50.25
	Proximal mediolateral width	17.09*
	Proximal anteroposterior width	14.46*
	Distal mediolateral width	22.15
	Distal anteroposterior width	19.97
**Phalanx I-1 (left)**	Length	64.8
	Proximal mediolateral width	20.9
	Proximal anteroposterior width	22.8
	Distal mediolateral width	18.6
	Distal anteroposterior width	17.8
**I-2 ungual (left)**	Length	60.6
	Proximal mediolateral width	16.6
	Proximal anteroposterior width	29.6
**MC II (left)**	Length	81.7
	Proximal mediolateral width	13.6*
	Proximal anteroposterior width	11.3
	Distal mediolateral width	14.1
	Distal anteroposterior width	12.8
**Phalanx II-1 (left)**	Length	31.1
	Proximal mediolateral width	11.3
	Proximal anteroposterior width	10.7
	Distal mediolateral width	9.4
	Distal anteroposterior width	8.6
**Phalanx II-2 (left)**	Length	35.9
	Proximal mediolateral width	12.2
	Proximal anteroposterior width	13.6
	Distal mediolateral width	11
	Distal anteroposterior width	9.5
**Phalanx II-3 ungual (left)**	Length	29.5*
	Proximal mediolateral width	8.5
	Proximal anteroposterior width	12.3
**MC III (left)**	Length	40.3*
	Proximal mediolateral width	3.3
	Proximal anteroposterior width	5.5
**Pubes**	Length (left)	355†
	Anteroposterior length of boot	158†
**Femur (right)**	Length	775
**Fibula (right)**	Length	341*
	Maximum width	98
**Metatarsal II (right)**	Length	128.94*
	Distal mediolateral width	44.05
	Distal dorsoventral height	44.58
**Metatarsal III (left)**	Length	309
	Proximal mediolateral width	53.17
	Proximal dorsoventral height	76.79
	Distal mediolateral width	56.3
	Distal dorsoventral height	40.61
**Metatarsal III (right)**	Length	124.3*
	Distal mediolateral width	63.18
	Distal dorsoventral height	43.29
**Phalanx II-1 (right)**	Length	95.25
	Proximal mediolateral width	36.7
	Proximal dorsoventral height	49.49
	Distal mediolateral width	37.76
	Distal dorsoventral height	31.18
**Phalanx II-2 (right)**	Length	64.32
	Proximal mediolateral width	31.75
	Proximal dorsoventral height	32.83
	Distal mediolateral width	28.86
	Distal dorsoventral height	26.84
**Phalanx II-3 ungual (right)**	Length	54.61*
	Proximal mediolateral width	21.85
	Proximal dorsoventral height	30.96
**Phalanx III-1 (right)**	Length	104.31
	Proximal mediolateral width	54.76
	Proximal dorsoventral height	42.98
	Distal mediolateral width	46.5
	Distal dorsoventral height	29.32
**Phalanx III-2 (right)**	Length	79.08
	Proximal mediolateral width	45.59
	Proximal dorsoventral height	31.96
	Distal mediolateral width	38.08
	Distal dorsoventral height	23.85
**Phalanx III-3 (right)**	Length	54.83
	Proximal mediolateral width	35.62
	Proximal dorsoventral height	24.71
	Distal mediolateral width	29.28
	Distal dorsoventral height	21.07
**Phalanx III-4 ungual (right)**	Length	58.12
	Proximal mediolateral width	22.75
	Proximal dorsoventral height	28.88
**Phalanx IV-1 (right)**	Length	73.39
	Proximal mediolateral width	30.96
	Proximal dorsoventral height	41.52
	Distal mediolateral width	33.44
	Distal dorsoventral height	26.61
**Phalanx IV-2 (right)**	Length	55.43
	Proximal mediolateral width	31.11
	Proximal dorsoventral height	31.82
	Distal mediolateral width	30.03
	Distal dorsoventral height	22.93
**Phalanx IV-3 (right)**	Length	54.06
	Proximal mediolateral width	28.97
	Proximal dorsoventral height	23.52
	Distal mediolateral width	20.97
	Distal dorsoventral height	16.15
**Phalanx IV-4 (right)**	Length	32.43
	Proximal mediolateral width	27.17
	Distal mediolateral width	26.7
**Phalanx IV-5 ungual (right)**	Length	42.74*
	Proximal mediolateral width	16.95
	Proximal dorsoventral height	23.04

* element exhibits breakage, measurement represents preserved dimension

† estimated measurement of broken element.

#### Provenance

The specimen came from a sandstone layer in a section of alternating sand- and mudstones (see S1 Fig) that make up the Cenomanian to Turonian aged Huincul Formation [25] exposed along the northern flank of the Meseta de la Rentería, Río Negro Province, Argentina (Fig 1B and 1C). Exact locality data are on file with the authors. Permission was obtained by the senior author for this study from the Agencia Cultura de Río Negro, and complies with all relevant regulations.

#### Differential diagnosis

*Gualicho shinyae* is distinguished by a unique combination of character states, which otherwise optimize as derived traits of very disparate theropod groups (see Description and Discussion). Posterior dorsal vertebrae very elongated and with slit-like pneumatic openings; scapular blade narrow with sinuous rostral margin marked by a shallow notch between the acromion process and blade; forelimb foreshortened with reduced muscle attachments and articulations and functionally didactyl; first and second metacarpals co-ossified proximally, third metacarpal reduced to a splint; pubes with little or no pubic apron and blade-like boot; femur with mediodorsally inclined head; reduced femoral distal condyles; fibula with large fossa and accessory flange on proximocaudal corner; ridge-like m. iliofibularis tubercle of fibula; third metatarsal with expanded proximal articulation with posterior edge wider than rostral edge (antarctometatarsal condition [8]); pedal unguals with single claw sheath grooves that define small spur or tuber near proximal end.

#### Etymology

*Gualicho*, a Spanish name derived from the Gennaken (günün-a-künna or northern Tehuelche language) *watsiltsüm*, for a goddess who was considered the owner of animals and later, following the introduction of Christianity, reinterpreted as a demonic entity. She is now considered a source of misfortune by rural settlers (gauchos) of the Southern Cone. The name was chosen to reflect the difficult circumstances surrounding the discovery and study of the specimen, and its contentious history following excavation. The specific name honors Ms. Akiko Shinya, Chief Fossil Preparator at the Field Museum, for her many contributions to paleontology including discovery of the holotype of *Gualicho* on February 13th, 2007 (see S1 Fig).

### Description and comparisons

#### Axial column

**Fig 2:**
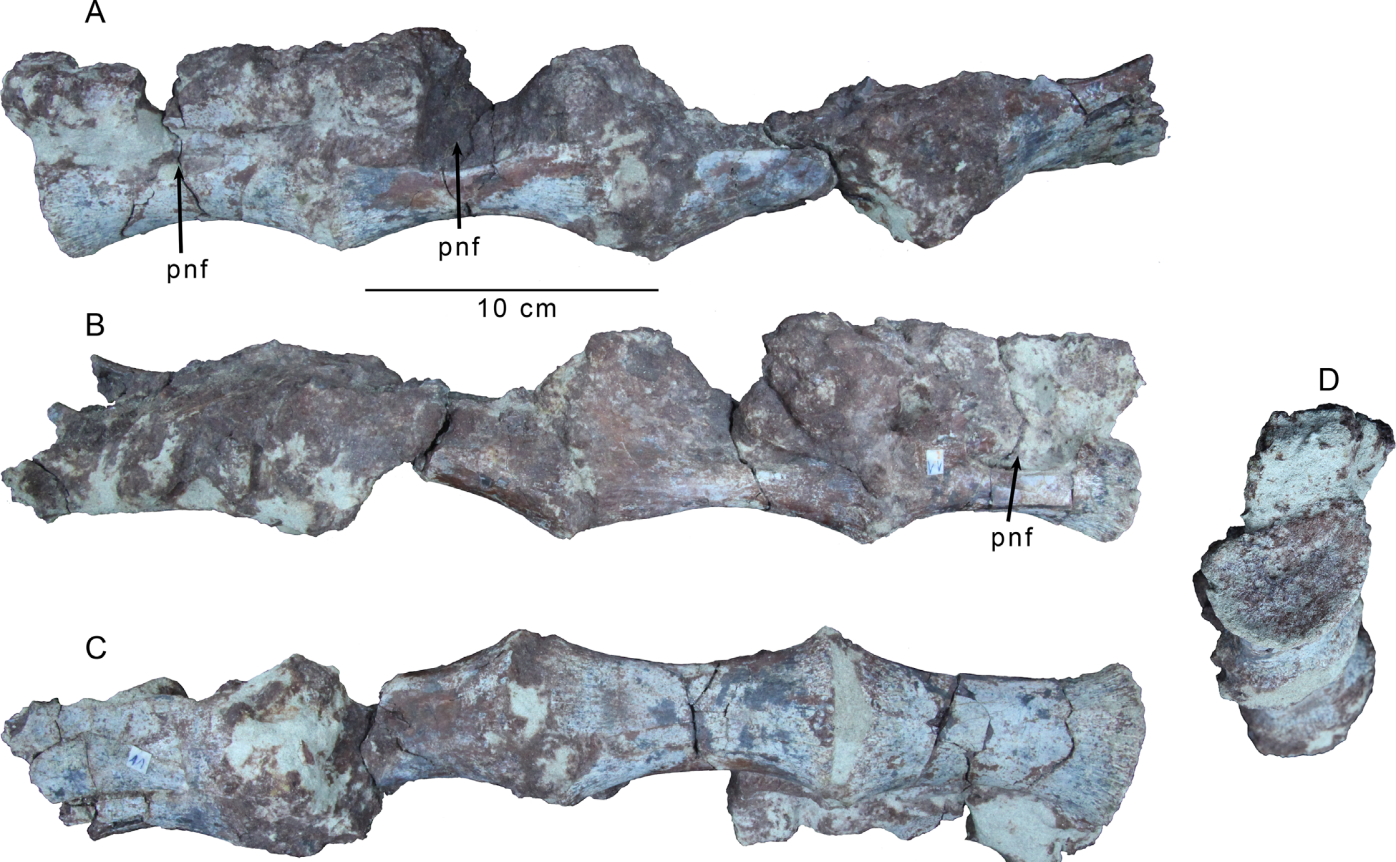
Articulated dorsal vertebral centra of *Gualicho shinyae*. A series of articulated posterior dorsal vertebral centra of the holotype specimen of *Gualicho shinyae* (MPCN PV 0001) in (A) left lateral, (B) right lateral, (C) ventral, and (D) anterior views. Abbreviation: pnf, pneumatic foramen.

Three dorsal centra are preserved in articulation, though the last one is missing the posterior half of its centrum (Fig 2). The absence of both parapophyses and ventral keels suggest they are from the caudal section of the dorsal series. The articular facets are flat, and the rims of the facets exhibit distinct longitudinal striations around the entire rims (Fig 2C), which are often present in the posterior dorsals of theropods. The centra are spool-shaped with elliptical articular faces, and are slightly compressed dorsoventrally. The centra are very elongate, roughly 2.5 times as long as the articular facets are dorsoventrally high. Such proportions are unusual among theropods, but are approached in some coelophysoids [26], ceratosaurs like *Masiakasaurus* [27] and *Elaphrosaurus* (MB.R. unnumbered), and also in the megaraptoran *Siats* [28]. Poorly preserved pneumatic openings are present on all three centra (Fig 2B). They are extremely elongate and slit-shaped, being dorsoventrally shallow, yet extending axially along the spool-portion of the centrum body. The left pneumatic opening of the first vertebra in the series is the best preserved, and indicates that the openings are confined to the centrum body, but rims are difficult to make out on the other elements. Unfortunately the poor preservation does not allow for an assessment of their depth, nor whether they deeply invade the centra. Dorsoventrally narrow pneumatic openings are observed on the dorsal vertebrae of some carcharodontosaurians such as *Siats* (FMNH PR 2716) and *Aerosteon* [29] (MCNA-PV-3137), but are absent in non-abelisaurid ceratosaurs with elongate dorsal centra such as *Spinostropheus* [30] and *Masiakasaurus* [27], as well as in coelophysoids [31], and other outgroups. A few fragments of bone that are likely from the neural arch of the first vertebra in the series are still connected by matrix, but little detail regarding their morphology can be discerned. An isolated partial centrum of another posterior dorsal is preserved, but was crushed considerably dorsoventrally. This element exhibits a tight fit with the block of three centra and constitutes the fourth element in the series. It also bears an elongate, slit-like pneumatic foramen (Fig 2B), though this is partially obscured by taphonomic distortion.

**Fig 3:**
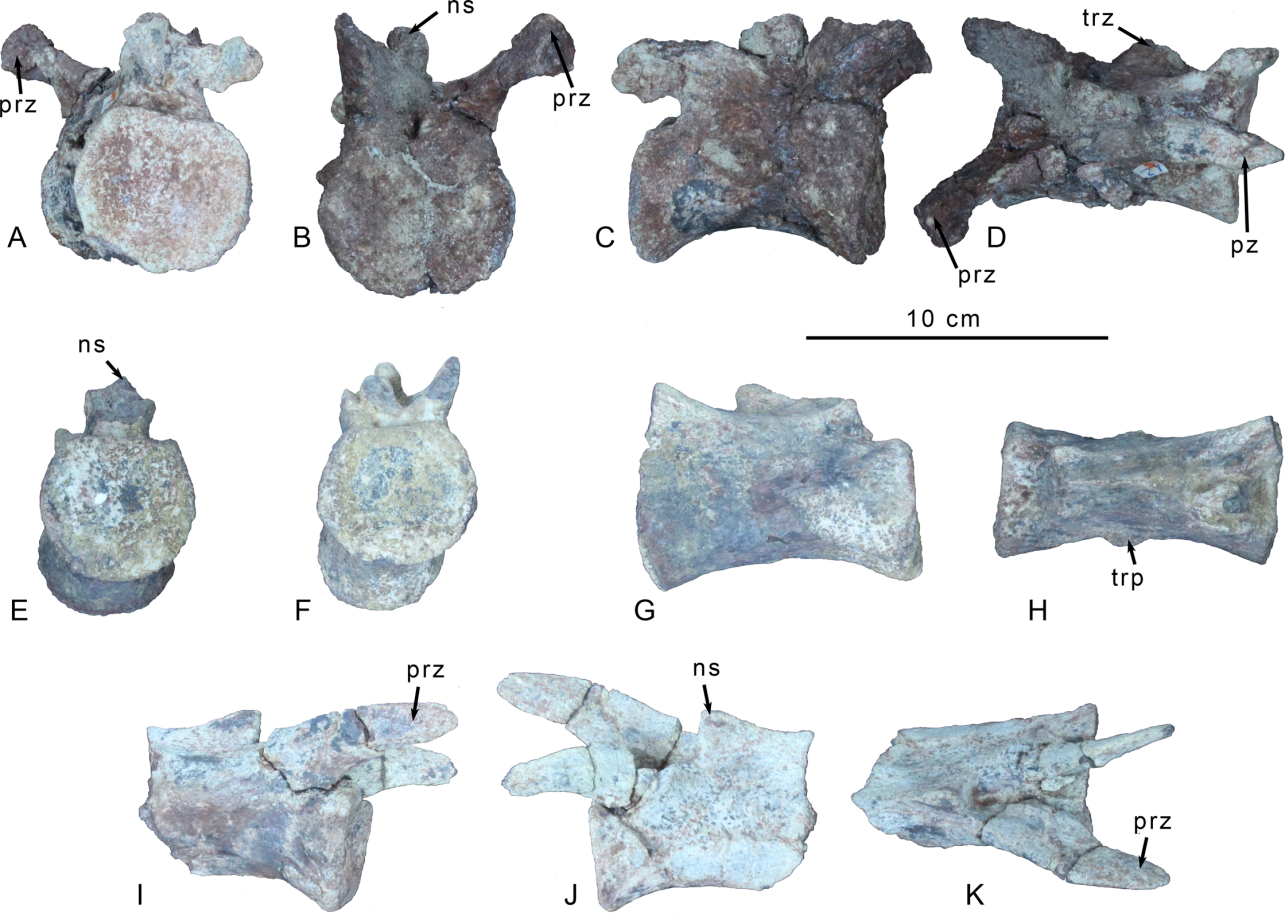
Preserved caudal vertebrae of *Gualicho shinyae*. Three mid-caudal vertebrae of the holotype of *Gualicho shinyae*. Anteriormost caudal in (A) posterior view, (B) anterior view, (C) right lateral and (D) dorsal views. Middle of the three caudals in (E) posterior, (F) anterior, (G) left lateral, and (H) ventral views. Posteriormost of the three caudals in (I) right lateral, (J) left lateral, and (K) dorsal views. Abbreviations: ns, neural spine; prz, prezygapophysis; pz, postzygapophysis; trp, transverse process.

Three caudal vertebrae from the middle of the tail are preserved (Fig 3). The articular facets are circular in end view and concave and the centra are spool-shaped and elongate, varying from about 1.5 to 2.0 times as long as the dorsoventral height of the articular facets. No sulci or ridges are observed on the ventral faces of the centrum bodies in the first two caudals. However, the last caudal, which is also the most axially elongated of the three, bears a faint midline ventral sulcus that is confined to the anterior half of the centrum body.

The largest of the three caudals is also the most complete. It retains transverse processes in the form of axially elongated, elliptical projections on the sides of the neural arch, slightly posterior to its midpoint, indicating it is close to or at the transition point (Fig 3A–3D). The prezygapophyses are stalked and project well beyond the anterior articular facet, further than the postzygapophyses, which only extend slightly past the posterior articular facet. The prezygapophyses are incomplete distally, and are angled anterodorsally rather than anteriorly. Stout ridges extend from the posterior base of the neural spine out to the tips of the postzygapophyses, which are canted with articular facets facing ventrolaterally. A strong ridge of bone also connects the lateral edge of the postzygapophysis to the middle of the lateral face of the neural arch. Only the posterior opening of the neural canal is visible and is rectangular and slightly wider than tall. A small depression is present dorsal to the neural canal, between the bases of the medial edges of the postzygapophyses. The base of a short neural spine is present, but is abraded and broken posteriorly.

Another caudal (Fig 3E–3H) exhibits even more reduced transverse processes, which are represented by low ridges on the sides of the neural arch. The neural spine is shallow, rectangular and axially elongate, unlike the tall, strap-like spines of many ceratosaurs including *Ceratosaurus* (UMNH VP 5278), *Masiakasaurus* [27] and *Carnotaurus* [32] (MACN-CH 894), but similar to the basal ceratosaur *Elaphrosaurus* (MB.R. unnumbered). The dorsal border of the spine has a weakly concave dorsal margin, giving it a saddle-shaped appearance in lateral aspect, though not to the degree that it appears bifid, as in e.g., *Allosaurus* [33]. The bifid condition is observed in many basal tetanuran lineages and is potentially a synapomorphy of a monophyletic Carnosauria [26]. Both pre- and postzygapophyses are broken in this specimen, but a low ridge spanning across the lateral face of the arch connects the base of the prezygapophysis to that of the postzygapophysis on each side.

The third caudal (Fig 3I–3K) is missing the posterior half of the centrum and postzygapophyses. The neural arch bears no trace of transverse processes suggesting this element represents a posterior caudal. The indented dorsal margin of the rectangular neural spine is below the level of the dorsal edges of the prezygapophyses. The prezygapophyses are relatively short and lobate in lateral aspect, and are significantly shorter than the length of the centrum.

#### Gastral basket

**Fig 4:**
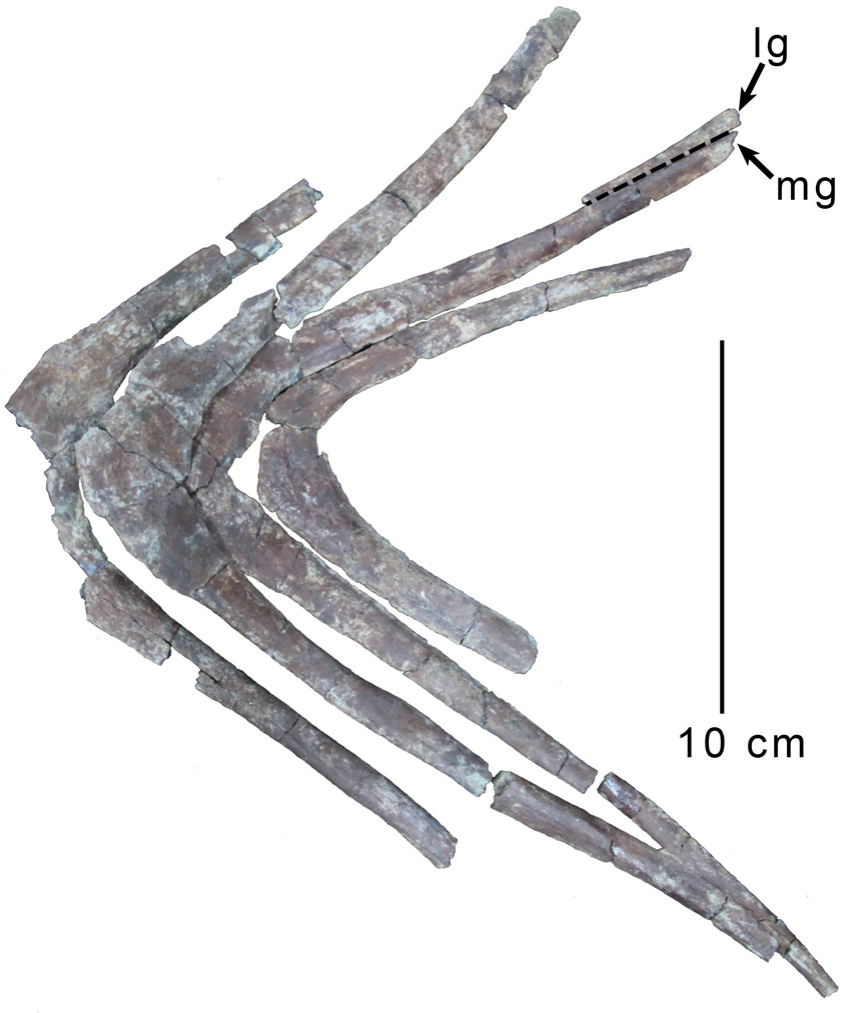
Posterior gastral arches of *Gualicho shinyae*. Last four preserved gastral arches of the holotype specimen of *Gualicho shinyae* (MPCN PV 0001) in dorsal view. Abbreviations: lg, lateral gastralia; mg, medial gastralia.

A near-complete and articulated gastral basket comprising 16 or 17 gastral rows was collected with the holotype. As in carcharodontosaurids [2], megaraptorans [29], and some other theropod groups [34], multiple arches are fused at the midline. At least six arches exhibit midline fusion in MPCN PV 0001, with fusion between elements observed in one anterior arch, and also in the five most posterior arches (Fig 4). These last five fused arches exhibit a progressively more acute angle between their rami posteriorly suggesting they are approaching the pubic boot. Notably, midline gastralia from the posterior portion of the gastral series found in contact with the pubic boot of *Deltadromeus* (SGM-Din 2) do not appear to be fused.

The rostralmost gastralia are thicker in girth than more posterior ones and also meet at a much shallower angle on the midline, as is typical for theropods [34]. More posterior elements are hooked at the midline where they form an expanded but flattened surface for fusion with the opposite medial element. Unlike some tyrannosaurid specimens [34], pronounced medioventral or mediodorsal facets for articulation with adjacent gastral rows are not observed in the holotype of *Gualicho*. The medial gastralia taper toward their lateral ends and some exhibit shallow grooves for articulation with lateral gastralia. Fragments of lateral gastralia are preserved in articulation with two of the medial rows, but none are complete so it is unknown whether lateral elements were shorter than medial ones, or vice versa. None of the gastral elements, whether fused or not, exhibit pneumatic openings such as those described in *Aerosteon* [29].

#### Pectoral girdle

**Fig 5:**
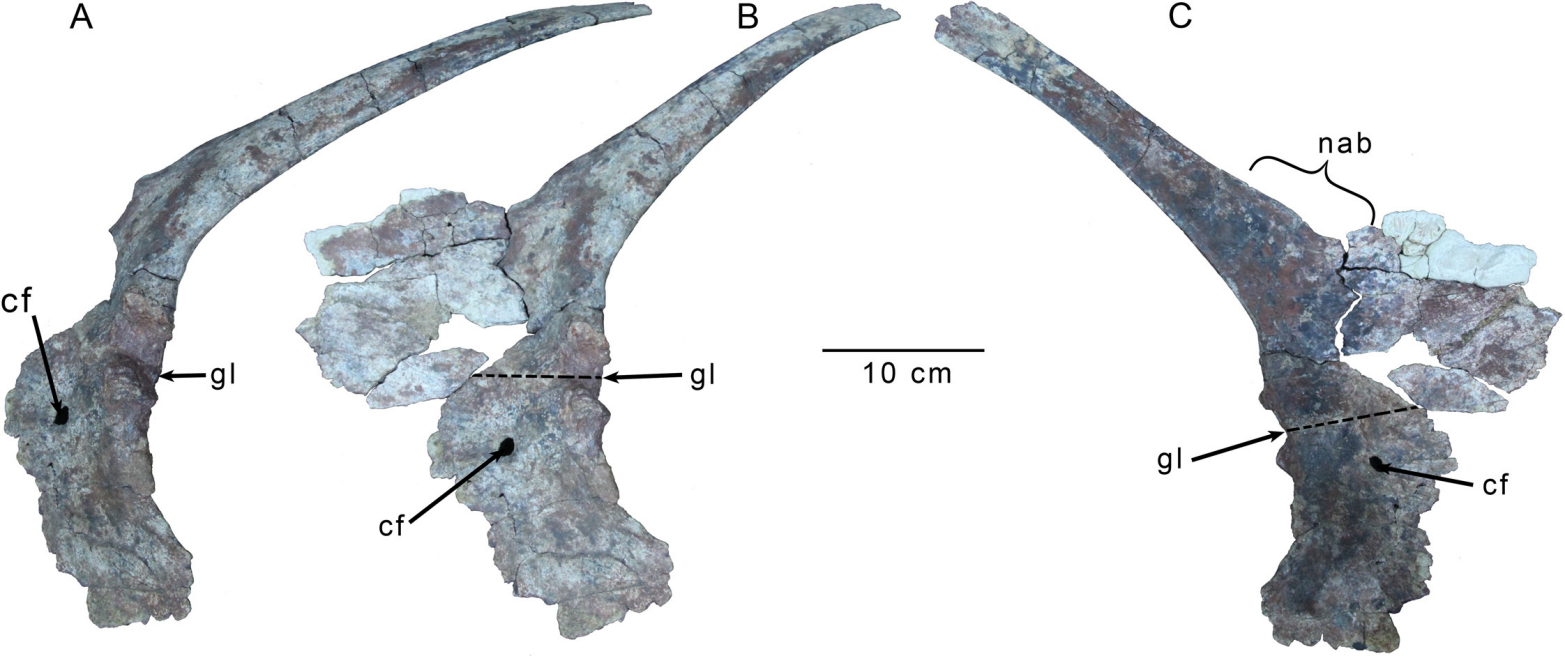
Left scapulocoracoid of *Gualicho shinyae*. Scapulocoracoid of the holotype specimen of *Gualicho shinyae* (MPCN PV 0001) in (A) posterolateral oblique (B) lateral, and (C) medial views. Dotted line indicates boundary between scapula and coracoid. Abbreviations: cf, coracoid foramen; gl, glenoid; nab, notch between scapular blade and acromion process.

The majority of the left scapula and coracoid are preserved (Fig 5), though the distal tip of the scapula is broken off, rendering its total length uncertain. The blade is strap-like, with a preserved scapular length more than 10 times the width at the narrowest point of the blade, a proportion similar to that observed in carcharodontosaurids including *Acrocanthosaurus* [35], and *Mapusaurus* [2], but also *Allosaurus* [7] and *Deltadromeus* (SGM-Din 2). Following Rauhut [26], Carrano et al. [7] found this elevated ratio to be a synapomorphy of some carcharodontosaurids, and possibly also Allosauridae, as well as of Coelurosauria. In contrast to these taxa, however, the blade appears short and less than twice the length of the acromion-glenoid distance in *Gualicho*, resembling *Deltadromeus* (SGM-Din 2) and *Masiakasaurus* [27], though the dorsal-most portion of the blade is not preserved in *Masiakasaurus* [27]. Unlike most tetanurans [26], the blade does not exhibit a subequal width throughout most of its length, and rather appears to taper distally from its base as in *Masiakasaurus* [27], *Limusaurus* [36] and *Deltadromeus* (SGM-Din 2). The blade is weakly convex laterally throughout its length implying low curvature of the rib cage. The lateral surface is weakly rounded while the medial surface is almost completely flat, and the ventral edge is slightly thicker than the dorsal edge. Near the base of the blade, the dorsal edge expands dorsally, but then arcs weakly back ventrally adjacent to the base of the expanded acromion process (Fig 5C). This sinuous margin creates a weak, rostrocaudally elongate flange along the dorsal edge that is separated from the base of the acromion process anteriorly by a broad and shallow indentation along the dorsal margin. A sinuous dorsal margin of the scapula adjacent to the acromion process defining a low flange is also observed in the African theropod *Deltadromeus* (SGM-Din 2), which also shares the presence of a relatively short, narrow, and distally tapering scapular blade with *Gualicho*. The scapula of *Limusaurus* [36] exhibits a deep, semicircular embayment of the rostral edge of the scapula at the transition between the acromion process and blade. Rostral to this indentation, the scapular margin expands smoothly dorsally to define the acromion process. The angle between the acromion process and the scapular blade is oblique, in contrast to the derived, perpendicular orientation seen in Allosauria and Coelurosauria [7]. Only the very base of the acromion process retains a natural edge, with the rest of the edges broken. However, the preserved edge is extremely thin, and likely did not continue much further, so that the outline of the preserved process is close to its original shape. The acromion process appears to have been shallow as in *Deltadromeus* (SGM-Din 2), *Giganotosaurus* (MUCPv-Ch 1), *Mapusaurus* [2], and *Acrocanthosaurus* [35], but not *Megaraptor* (MUCPv 341), *Allosaurus* [33], and *Sinraptor* [37]. The base of the coracoid process of the scapula is preserved with the scapular blade, but the glenoid portion is broken off and preserved with the coracoid.

The scapula and coracoid are fused but not completely co-ossified, and a line of fusion can still be discerned on both sides, though it is more visible on the medial side (Fig 5B and 5C). The scapula contributes about two thirds of the glenoid articulation, whereas the coracoid contributes the remaining third (Fig 5A). The articular surface of the glenoid is angled outward slightly, such that it faces ventrolaterally. A small lip is formed by the scapula and coracoid at the dorsal and ventral margins of the glenoid, respectively. These lips are not laterally everted, and instead project caudally (Fig 5B). Similar lips are present in many ceratosaur taxa including *Elaphrosaurus* (MB.R. unnumbered), *Masiakasaurus* [27], and *Carnotaurus* (MACN Ch 895), but also are observed in the megaraptoran *Aerosteon* [29] (MCNA-PV-3137). Unlike *Elaphrosaurus* (MB.R. unnumbered), the glenoid lips do not merge to form a rim around the entire glenoid, but rather are restricted to the ventral and dorsal limits of the articulation.

A large, oval coracoid foramen is present about eight centimeters anterior to the glenoid (Fig 5A–5C). The majority of the coracoid is weakly convex laterally, with the exception of a small area just dorsal to the coracoid foramen and anterior to the suture between the scapula and coracoid that is shallowly depressed. A coracoid (= 'biceps') tubercle is absent, as is the case in *Masiakasaurus* [25], *Deltadromeus* (SGM Din2), and many Megalosaurians [7], but in contrast to the condition in most tetanurans, which possess an oblique ridge-like tubercle [7]. The posteroventral process of the coracoid is hooked and extends far ventral to the glenoid, to a degree similar to that seen in *Deltadromeus* (SGM-Din 2), and *Megaraptor* (MUCPv 341). A well-developed posterovental process is only present in *Masiakasaurus* [27] and *Elaphrosaurus* (MB.R. unnumbered) within Ceratosauria, and in these two taxa, the process is not as extensive as in *Gualicho*. Within Tetanurae, a pronounced posteroventral process is absent in basal members such as '*Dilophosaurus*' *sinensis*, *Torvosaurus*, *Megalosaurus*, and *Yangchuanosaurus hepingensis*, but is a synapomorphy of Allosauria [7]. Its posterior edge below the glenoid is also everted slightly laterally, much like the glenoid articulation. This everted edge is widest just below the glenoid, and it thins ventrally along the posteroventral process. This area of the posteroventral process also lacks the distinct fossa (Fig 5A) that is present in megaraptoran taxa [38].

#### Forelimb

**Fig 6:**
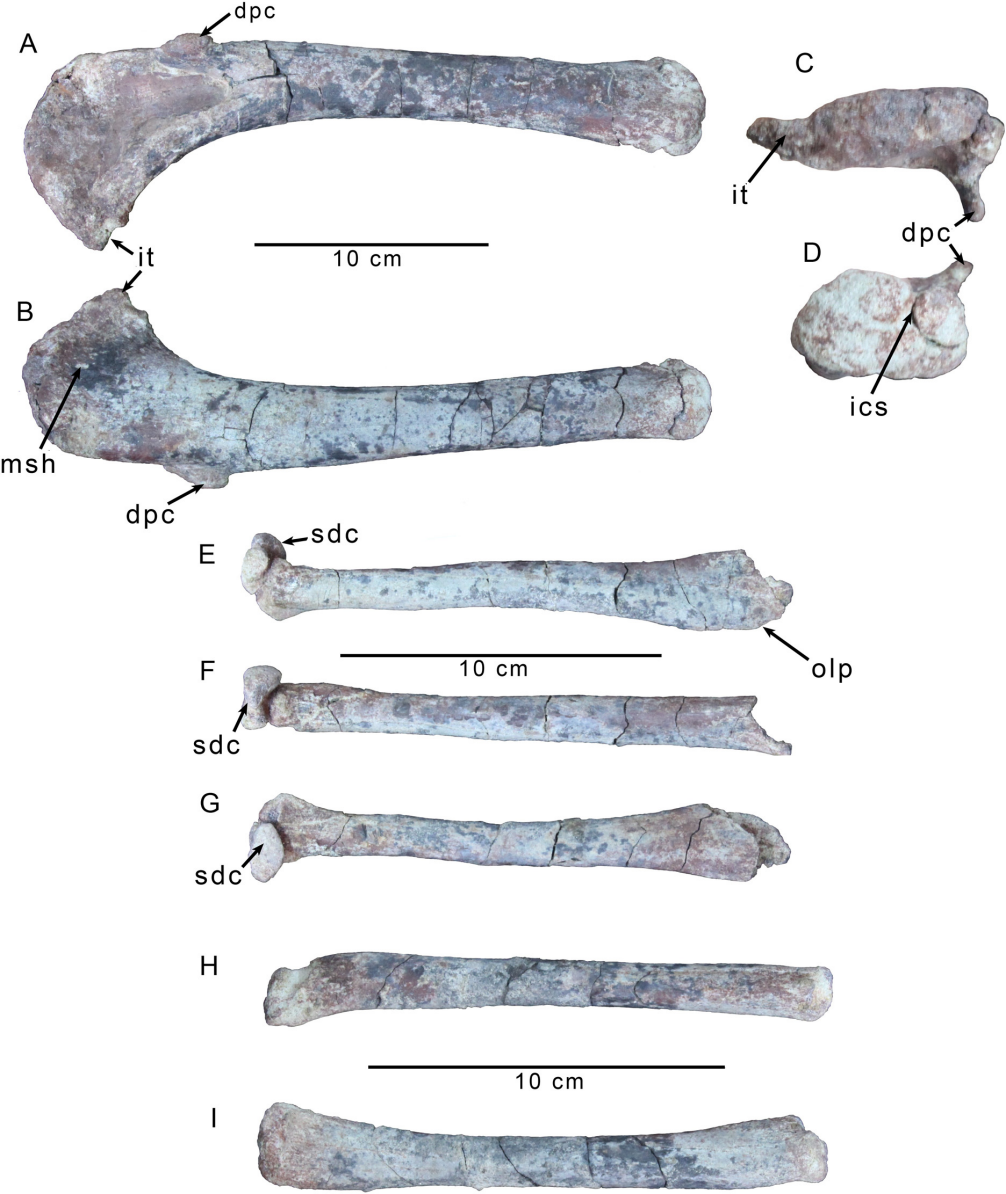
Forelimb elements of *Gualicho shinyae*. Left humerus of the of the holotype specimen of *Gualicho shinyae* (MPCN PV 0001) in (A) anterior, (B) posterior, (C) proximal, and (D) distal views. Left ulna and attached semilunate distal carpal of the holotype specimen of *Gualicho shinyae* (MPCN PV 0001) in (E) lateral, (F) posterior, and (G) anterior views. Left radius of the holotype specimen of *Gualicho shinyae* (MPCN PV 0001) in (H) lateral, and (I) medial views. Abbreviations: dpc, deltopectoral crest; ics, intercondylar sulcus; it, internal tuberosity; msh, scar for insertion of m. scapulohumeralis; olp, olecranon process base; sdc, semilunate distal carpal.

Parts of both forelimbs were collected with MPCN PV 0001, including a complete left forelimb. The right forelimb is represented by the radius and ulna. The humerus is almost straight with only a slight laterally convex bow (Fig 5A and 5B). The distal condyles are twisted laterally about 15 degrees relative to the humeral head (Fig 6C and 6D), which is mediolaterally elongate, unlike the spherical humeral head of many ceratosaurs [26] including *Deltadromeus* (SGM-Din 2) and *Elaphrosaurus* (MB.R. unnumbered). There is a very weak cleft between the head and the pointed internal tuberosity (Fig 6B). The internal tuberosity is distinctly thinner than the head in proximal view. There is no evidence of a deep, longitudinal furrow on the caudomedial side of the proximal humerus, as is present in *Australovenator* [39], *Fukuiraptor* [40], *Chilantaisaurus* [41], and *Megaraptor* [11]. Although this feature has also been suggested to be present in tyrannosaurids [12], this is based on misinterpretation of a pathology in one specimen of *Tyrannosaurus rex* [42, 43] (FMNH PR2081). Two broad depressions are present on the anterior face of the humerus, though these are slightly accentuated by crushing of the element. The proximal depression is just below the humeral head and is mediolaterally elongate, extending across the breadth of the bone. The distal depression is more elongate and situated in the middle of the bone just medial to the deltopectoral crest. The deltopectoral crest is set perpendicular to the long axis of the humeral head (Fig 6A and 6B), as in a majority of theropods with the exception of some megalosaurians that have an anterolaterally directed deltopectoral crest [7]. It is proximodistally elongate and spans approximately one quarter the length of the humerus. Among theropods, a deltopectoral crest with such a limited proximodistal extent is only seen in *Deltadromeus* (SGM Din-2) and Ornithomimosauria [26], although *Masiakasaurus* [27] and *Elaphrosaurus* (MB.R. unnumbered) approach this condition. It tapers to a thin (and abraded) edge proximally, but its apex is thickened mediolaterally into a lobate tuberosity that is a proximodistally elongate ellipse in anterior aspect. A distinctly offset and lobate apex on the deltopectoral crest is widespread among allosauroid species including *Acrocanthosaurus* [35], *Neovenator* [44], and *Fukuiraptor* [40], but is not observed in megalosauroids [45], ceratosaurs (e.g., *Ceratosaurus* UMNH VP 5278), coelophysoids (*Dilophosaurus* UCMP 37302), and coelurosaurs (e.g., *Compsognathus* MB.R.2003.2; *Guanlong* IVPP V14531). There is a small, circular divot on the posterior surface of the humerus (Fig 6B), just below the midpoint of the head that is located in a topologically identical position to a large fossa on the humerus of *Acrocanthosaurus* (NCSM 14345). This part of the humerus likely serves as an insertion point for m. scapulohumeralis posterior, as in modern birds [46, 47].

The humeral shaft is relatively straight and cylindrical below the level of the deltopectoral crest, and lacks the distinct curvature seen in many tetanurans including *Piatnitzkysaurus* (MACN-CH 895), *Allosaurus* [33], and *Torvosaurus* [48]. There is a broad but very shallow brachial fossa on the anterior face of the distal end of the humerus that is far less developed than in most tetanuran taxa. The distal condyles exhibit no mediolateral expansion, nor are any epicondylar tubers present. The distal articular end is somewhat flattened and slightly abraded. There is no strong cleft or separation into distinct condyles, except for a small notch on the rostral aspect of the distal end (Fig 6D). The lateral condyle is much more robust and expanded anteroposteriorly than the medial condyle.

The proximal left ulna is broken, but the right element retains most of a modestly sized, rounded olecranon process, the medial portion of which is broken away. The proximal end is only slightly caudally expanded relative to the shaft, which is straight unlike the strongly curved ulna seen in most tetanurans such as *Allosaurus* [33] and *Concavenator* [49]. The midshaft is subcircular in cross section, and there are faint striations extending down the anterior to anteromedial surface of the ulnar shaft, which may mark the separation between the origin of m. abductor pollicis longus medial to the striations from the insertion of m. anconeus lateral to the striations [47]. The distal end is weakly expanded mediolaterally. As in most tetanurans, the distal articulation is flattened, unlike the derived, convex condition observed in abelisaurids [8].

The radial shaft is comparable in diameter to the ulna (Fig 6E–6I). The distal end is slightly expanded and medially offset from the main axis of the shaft. The distal articular surface is weakly triangular in distal aspect. Longitudinal striations extend throughout the shaft, but are faint and not well developed. There is a small, one centimeter long linear tuberosity located approximately five centimeters from the distal end of the radius, that projects laterally slightly above the shaft. The proximal face of the radius is better preserved on the right element and is rounded and globular, with a slight divot on one edge.

Two carpals are preserved with the left forelimb. The larger of the two is the compound semilunate carpal and is attached by matrix, though not co-ossified with, the left ulna (Fig 6E–6G). Its distal surface was discovered in articulation with the proximal articular surfaces of metacarpals I and II. It has a semilunate shape in lateral view, with a convex proximal surface and a flatter distal one. The proximal aspect is partly covered by the matrix connecting it to the ulna, but the exposed rostral section reveals a broad, shallow sulcus as in *Allosaurus* [33, 50], and *Sinraptor* [37]. The distal articulation is elliptical in end view with notches at the rostral and caudal ends that correspond to the termini of the longitudinal sulcus along the proximal surface. Its surface is kinked into a smaller rostral and a larger caudal area, again resembling the condition in *Allosaurus* [33] and other neotetanurans [7]. The medial face of the carpal is much smaller than the lateral one.

**Fig 7:**
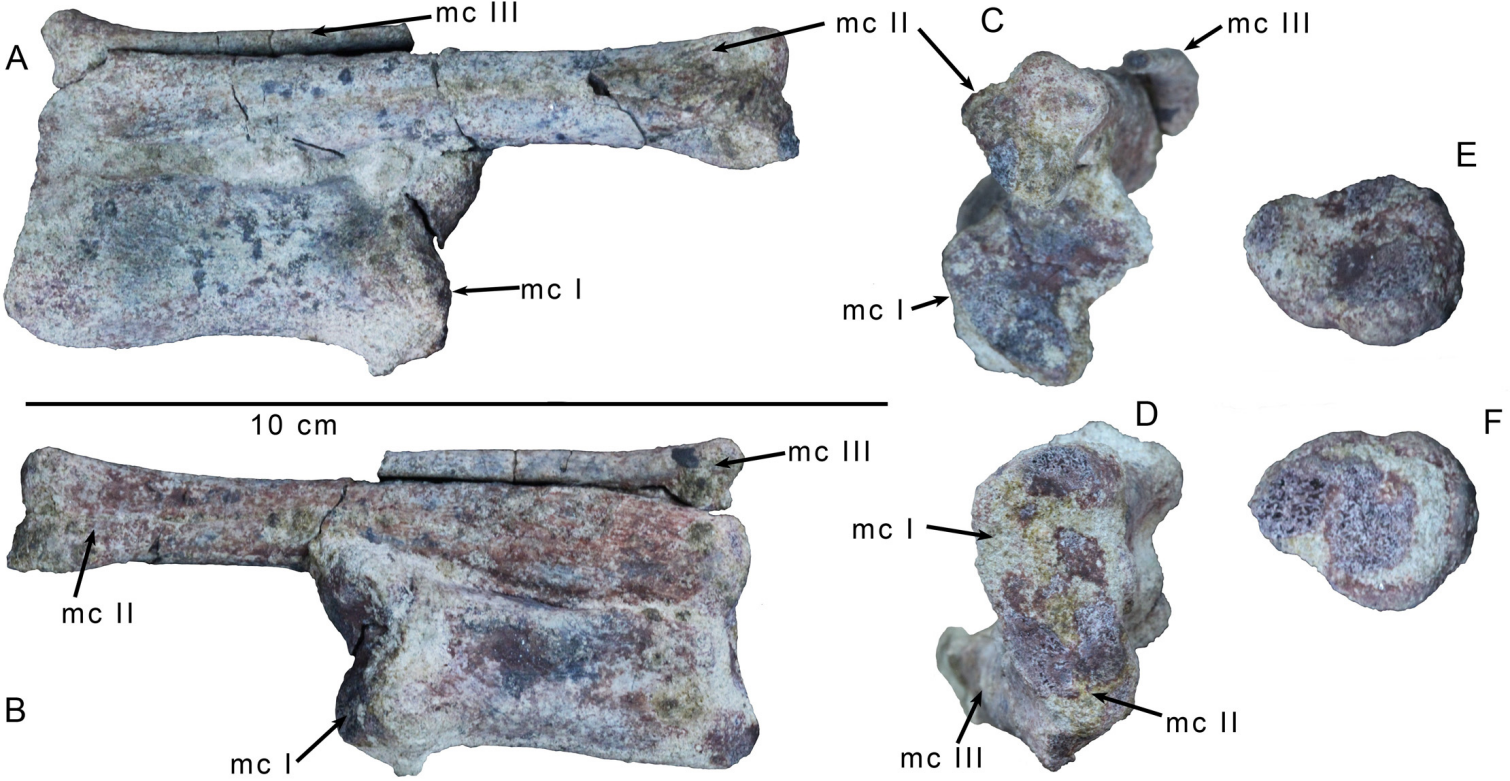
Wrist and palm elements of *Gualicho shinyae*. Articulated metacarpals I-III of the holotype specimen of *Gualicho shinyae* in (A) dorsal, (B) ventral, (C) distal, and (D) proximal views. Scapholunare of the holotype specimen of *Gualicho shinyae* in (E) proximal and (F) distal views. Abbreviation: mc, metacarpal.

The second carpal is smaller and flatter than the first (Fig 7E and 7F). In distal view, it has an irregular elliptical outline that is weakly constricted. Because no element was found lateral to this carpal, it is likely the scapholunare. It does not resemble the large kidney shaped scapholunare reported in *Australovenator* [39], but the presence of a slight notch or constriction is similar to *Allosaurus* [50], and *Acrocanthosaurus* [35].

The unusual, didactyl manus of *Gualicho* is dominated by a robust metacarpal I that is about twice the mediolateral width of metacarpal II (Fig 7A and 7B). The proximal end bears a slightly expanded, shallowly concave articular facet that is rectangular rather than triangular in end view (Fig 7D). The dorsal border of the articulation is straight, but the ventral (palmar) rim is gently concave, though not to the degree observed in either *Australovenator* or *Acrocanthosaurus*. An expanded proximomedial process as occurs in *Australovenator* [39] is absent. The width across the distal condyles is greater than that of the proximal articulation (Fig 7B). The distal articulation is not twisted relative to the proximal surface. Of the two hemicondyles, the medial one is much deeper in distal aspect, though both hemicondyles are roughly equal in mediolateral width (Fig 7C). A shallow sulcus separates them distally. A small tuber is present on the medial surface of the medial hemicondyle in place of a collateral ligament pit, though it is uncertain whether this represents an autapomorphy, or perhaps, a pathology.

Metacarpal II is proximally fused with metacarpal I and their shafts are closely appressed to each other throughout (Fig 7A and 7D). The proximal surface of MC II is very abraded, but is slightly angled relative to that of MC I. Unlike carcharodontosaurian taxa, such as *Acrocanthosaurus* [35] and *Megaraptor* [51], the base of metacarpal II is not broadly expanded, and the shaft is cylindrical and relatively slender overall, especially when compared with metacarpal I (Fig 7A and 7B). There is a broad but very shallow fossa on the anterior surface of the shaft, just below the proximal end. A teardrop-shaped extensor fossa is also present distally on the anterior surface. The distal hemicondyles are roughly equal in size and mediolateral width, though the medial condyle is distinctly deeper than the lateral one.

Metacarpal III is reduced to a thin splint probably lacking a distal articulation, as in tyrannosaurids (Fig 7A and 7B). Its proximal end is slightly expanded, but very abraded. The shaft is weakly elliptical in cross section, being slightly broader anteroposteriorly than mediolaterally. It is nearly straight with only a weak curvature in the anteroposterior plane. Metacarpal III is broken distally, but the preserved portion is just slightly shorter than MC I. The small cross section of bone at the distalmost preserved portion of MC III argues against the presence of a distal articulation for any phalanges.

**Fig 8:**
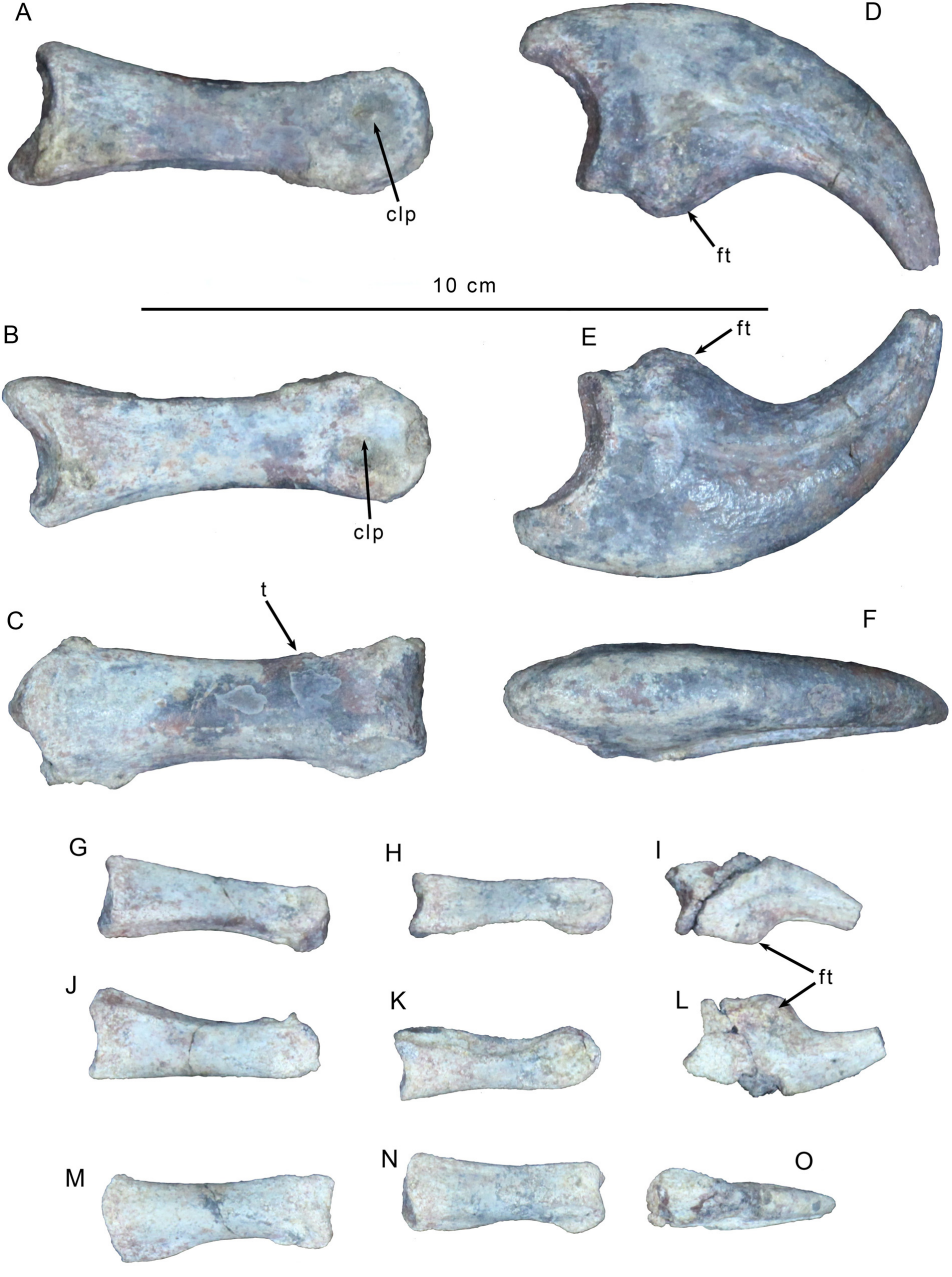
Left manual digits of *Gualicho shinyae*. Digit I phalanges in (A, D) medial, (B, E) lateral, and (C, F) dorsal views. Digit II phalanges in (G-I) medial, (J-L) lateral, and (M-O) dorsal views. Abbreviations: clp, collateral ligament pit; ft, flexor tubercle; t tuber.

As with the metacarpals, the phalanx and ungual of digit I are roughly twice as large as those of digit II (Fig 8). The ventral surface of I-1 is round and lacks a sulcus as is seen in megaraptoran theropods such as *Megaraptor* [52] and *Australovenator* [39; 53]. A tab-like ridge on the posterior edge of the proximal articular surface is canted slightly laterally, rather than being in the middle of the posterior edge. The distal articulation bears deep, symmetrical collateral ligament pits, and the hemicondyles are approximately equally developed (Fig 8A–8C). However, a distinct tuberosity is present on the posterior surface just proximal to the medial condyle that is not as well developed on the lateral condyle.

Ungual I-2 is slightly longer than phalanx I-1, though its distal tip is missing. The proximal articular surface is less than twice as high anteroposteriorly as broad mediolaterally, in contrast to the transversely narrow proportions that are a synapomorphy of some neovenatorids, including *Australovenator*, *Chilantaisaurus*, *Fukuiraptor*, and *Megaraptor* [7; 38]. The claw is highly recurved such that with the proximal articulation oriented vertically, the tip of the ungual reaches well below the flexor tubercle (Fig 8A and 8B). The length of the ungual is over twice as great as its height, similar to the condition present in *Baryonyx*, *Chilantaisaurus* [41], *Megaraptor* [52], *Suchomimus*, and *Torvosaurus* [54]. Single, symmetrical vascular grooves are present on both sides. The flexor tubercle is robust and mound-shaped, but less than half the height of the proximal articular facet.

Phalanx II-1 is slightly (~1cm) longer than both phalanges II-2 and II-3, but is shorter than all the metacarpals (Fig 8G–8O). In ventral view the proximal end of the shaft is asymmetric with the lateral edge more expanded than the medial one. The same asymmetry in the tuberosities above the distal condyles on the posterior face present in phalanx I-1 is also present here (i.e. with the medial tuberosity larger). Both collateral ligament pits are present but abraded. Phalanx II-2 has the same asymmetry in the development of the medial and lateral edges just below the proximal articular surface as in phalanx II-1. There is some amount of crushing on the posterior face of the bone in this area and just distal to it. Ungual II-3 is partially broken and missing its distal tip (Fig 8I, 8L and 8O). It is similar to ungual I-2 in having a broad proximal articulation and single vascular grooves on each side, but is notably less recurved.

#### Pelvic girdle

**Fig 9:**
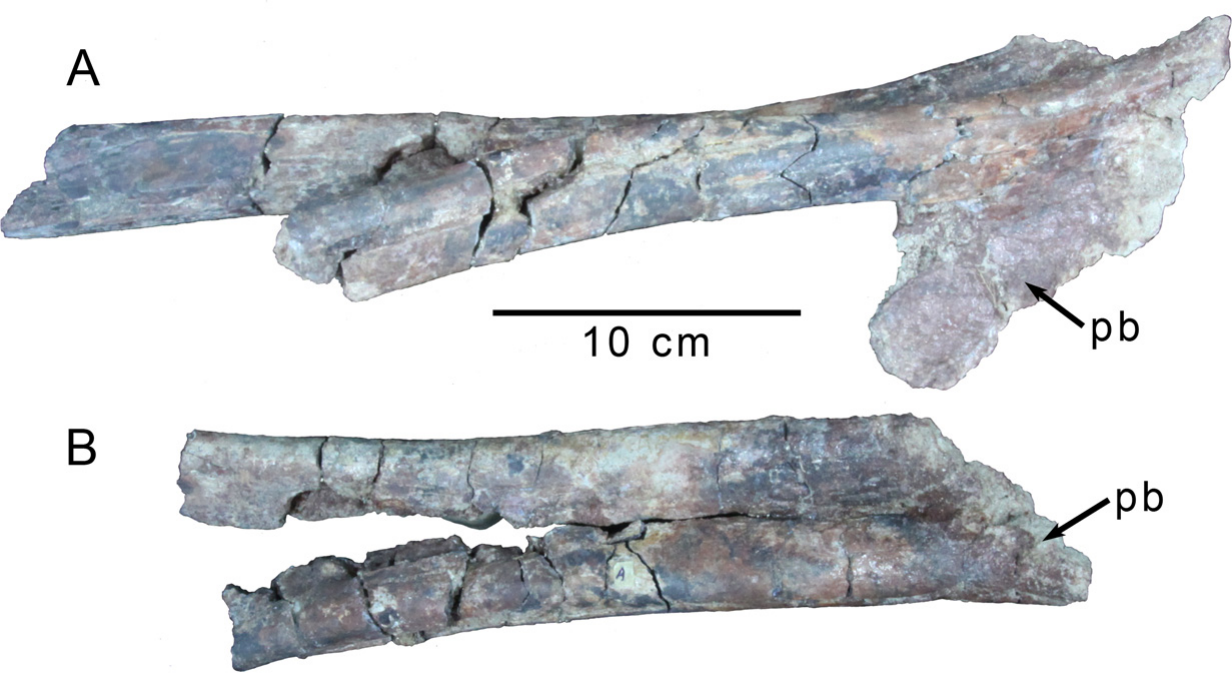
Distal pubes of *Gualicho shinyae*. Distal pubic shafts and pubic boot of the holotype specimen of *Gualicho shinyae* in (A) right lateral and (B) anterior views. Abbreviation: pb, pubic boot.

Only the distal pubes, including the boot, are preserved (Fig 9). A small portion of the proximalmost left pubic shaft is broken, but articulates cleanly with the larger piece. The shaft is robust and elliptical in cross section with the long axis oriented anterolaterally-posteromedially, and a narrow pubic apron extending from its posteromedial edge. The lateral face is round throughout its length. Although the medial edges of the shafts are broken, it appears that the pubic apron was open medially for the proximal half of the preserved elements, where the shafts are converging on each other in a broad V-shape. Further distally, where the pubic shafts are more parallel, the two sides contact along their posteromedial edges, leaving a deep but narrow rostral groove between the conjoined elements (Fig 9B). The distal portions of the pubes are conjoined medially as in all averostrans [7], but a distal pubic foramen, as present in tetanurans [7], is absent in *Gualicho*. As the pubic shafts converge they also twist laterally about their long axes, such that where the shafts contact they are now mediolaterally elongate ellipses in cross section. Though slightly distorted, the pubic shafts are relatively straight throughout their length, with only a slight anterior convexity (accentuated by the pubic boot) at their distal ends.

The edges of the pubic boot are poorly preserved, but it is clearly expanded both anteriorly and posteriorly, though the anterior expansion is only observable on the right pubis (Fig 9A). The boot is fully fused and compressed mediolaterally, and would not have been broadly expanded ventrally as seen in a number of tetanurans including *Giganotosaurus* (MCF Pv Ch 1), *Aerosteon* (MCNA-PV-3137), and *Torvosaurus* [48]. A mediolaterally compressed pubic boot with a narrow ventral edge is observed in *Deltadromeus* (SGM-Din 2; note that the element originally identified as the pubic boot [9] is actually the ischiadic symphysis [8]), and some coelurosaurs (e.g., *Ornithomimus* RTMP 95.110.1), and was recovered as a potential coelurosaurian synapomorphy [26]. The posterodorsal edge of the boot forms a sharp edge that diverges rostrally into two ridges, each one reaching a short distance up the caudal surface of a pubic shaft. A deep conical depression invades the dorsal aspect of the boot between the pubic shafts, but is closed off rostrally, resembling the condition described for *Masiakasaurus* [55], and also observed in taxa as diverse as *Deltadromeus* (SGM-Din 2) and *Coelurus* (YPM 1993).

#### Hind limb

**Fig 10:**
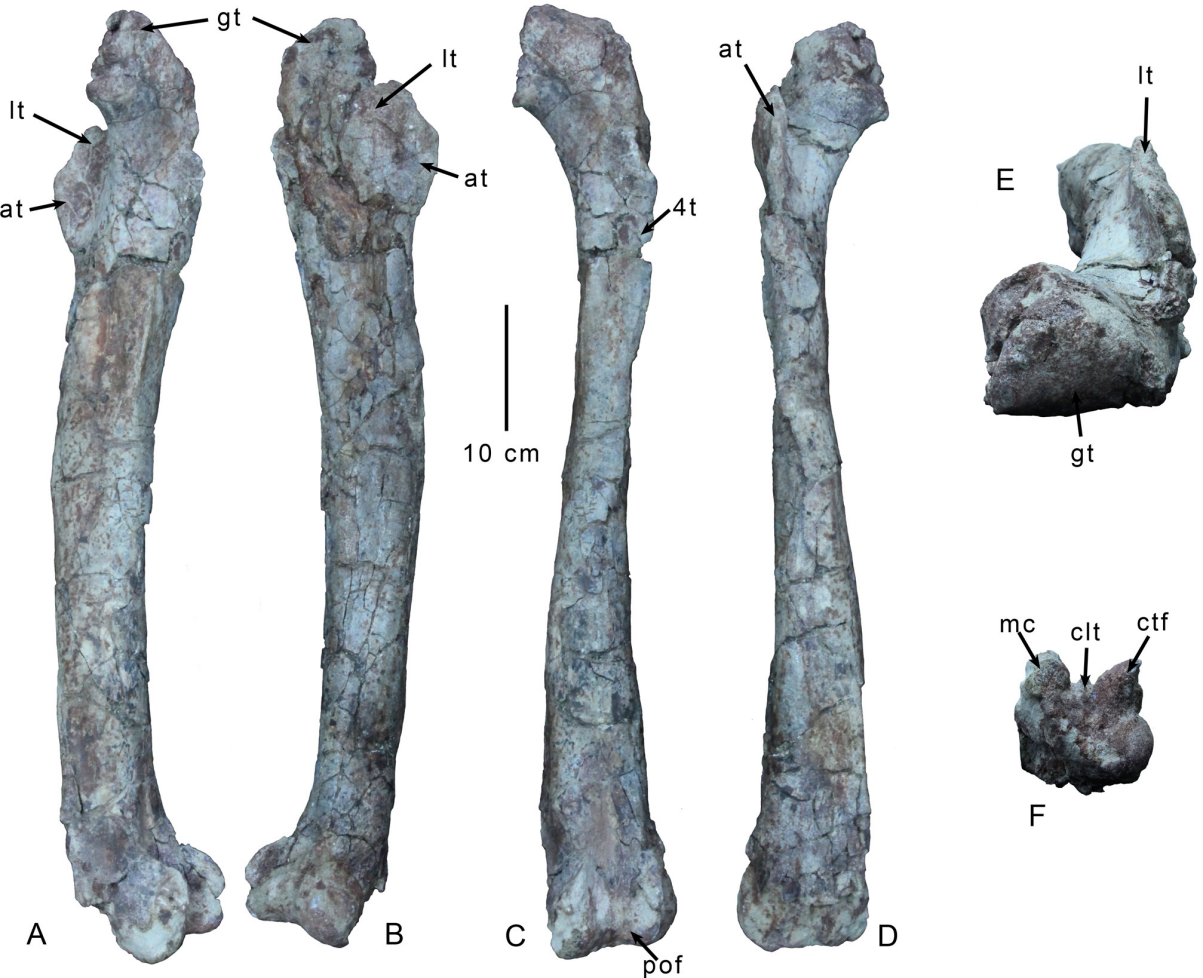
Right femur of *Gualicho shinyae*. Right femur of the holotype specimen of *Gualicho shinyae* in (A) medial, (B) lateral, (C) posterior, (D) anterior, (E) proximal, and (F) distal views. Abbreviations: 4t, fourth trochanter; at, accessory trochanter; clt, cruciate ligament tuber; ctf, crista tibiofibularis; gt, greater trochanter; lt, lesser trochanter; mc, medial condyle; pof, popliteal fossa.

The right femur of MPCN PV 0001 is almost complete (Fig 10), whereas the left is only represented by an extremely crushed distal end. The femoral head is incomplete, with the medial half of the head missing, precluding observations on the presence and form of the posterior sulcus. A proximal articular sulcus on the proximal surface of the head as is found in silesaurids, some basal sauropodomorphs and coelophysoids [56] is likely absent, as in most avetheropods [7], because at least the lateralmost extent of this sulcus should be visible on the preserved portion of the femoral head if it were present (Fig 10E). The femoral head is angled primarily medially relative to the orientation of the distal condyles, with only a minor anterior angle, though this impression may be accentuated by the missing medial portion of the femoral head. It also appears to be canted slightly proximally as well (Fig 10C), similar to the condition in *Deltadromeus* (SGM-Din 2), though to a lesser degree than in carcharodontosaurids [7, 57].

The femur bears an extensive lesser trochanter that is blade-like and broken at its proximal tip, but appears unlikely to have reached the level of the femoral head proximally (Fig 10A and 10B and 10D). An enlarged accessory trochanter projects from it at midheight (Fig 10A). Although large, it is not as prominent as in *Neovenator* [44]. The anterior trochanteric border slopes gradually into the femoral shaft below it. There is a very weak cleft between the proximalmost portion of the lesser trochanter and the femoral shaft. The fourth trochanter is reduced and projects weakly from the femoral shaft (Fig 10C). It is a low, proximodistally elongate ridge several millimeters thick that begins just below the level of the lesser trochanter and extends distally for about 12 centimeters. The femoral shaft is mediolaterally compressed and gracile, with a gentle, anteriorly convex curvature. The proximal portion of the shaft at the level of the fourth trochanter has been crushed on its lateral side. The anterior surface of the shaft is poorly preserved at the distal end, but the area preserved is very flat and appears to lack an extensive extensor fossa (Fig 10D), which is present in nearly all tetanurans [7] with the exception of the possible basal members *Cryolophosaurus* [58] (FMNH PR1821) and *Chuandongocoelurus* (CCG 20010). The base of a medial epicondylar crest is preserved, though most of the distal portion of the crest is broken. Based on the proximal portion of the crest, and the preserved distal part of the left femur, it is weakly developed and far less prominent than in, for example, *Coelophysis* [31] and *Liliensternus* (MB R. 2175). It is clear that the crest did not project far proximally, and was restricted to the distal one-fifth of the femur. There are relatively thick longitudinal striations on the medial surface of the femoral shaft in the area of the epicondylar crest that likely correlate with the origin of m. femorotibiales internus (medialis) [59].

The distal condyles are not mediolaterally expanded beyond the borders of the shaft (Fig 10F) unlike many basal tetanurans such as *Baryonyx* (NHMUK R9951), *Megalosaurus* [45], and *Sinraptor* [37]. A distinct and robust horizontal ridge of bone extends between the medial condyle and the crista tibiofibularis on the posterior side closing off the ventral end of the popliteal fossa, and may mark the insertion for the cruciate ligaments (Fig 10F). Such a ridge is present in a number of coelophysoids including “*Syntarsus*” *kayantakatae* [29] and ceratosaurs such as *Ceratosaurus* [60] (UMNH VP 5278), but is absent among tetanuran species [7]. A proximodistally elongate tuberosity projects about 2 cm posteriorly from the middle of this bridge, but is a widespread structure observed in numerous taxa including *Sinraptor* [37], *Acrocanthosaurus* [35], and *Deltadromeus* (SGM Din2). There is a small depression in the middle of the distal surface of the femur, just proximal to the cruciate bridge and between the proximal ends of the medial condyle and the crista tibiofibularis. A deep popliteal fossa is present on the posterior side of the distal femur. It creates a depression on the proximal side of the cruciate bridge and is deepest in this area, just between the proximal ends of the medial condyle and the crista tibiofibularis. The fossa extends up the femoral shaft about 12 centimeters before grading smoothly into the femoral shaft. The lateral condyle is bulbous and well rounded, projecting primarily laterally, but slightly anteriorly from the distal end of the femur (Fig 10C and 10F). The crista tibiofibularis is slightly less robust than the medial condyle. It is compressed strongly mediolaterally, particularly at its proximal end, which is blade-like. The proximal portion of the medial condyle is also compressed mediolaterally and its tip is similarly blade-like. The lateral face of the crista tibiofibularis is not circumscribed by a prominent groove as is observed in *Dilophosaurus* (UCMP 37302), some other coelophysoids [31], and *Masiakasaurus* [55].

The distal portion (~30cm) of the left femur is also preserved, though its entire anterior side has been crushed and sheared medially, and both condyles are crushed and extremely abraded. The medial edge is slightly distorted, but well preserved, and confirms that the medial epicondylar ridge is very reduced and not flange-like in morphology.

**Fig 11:**
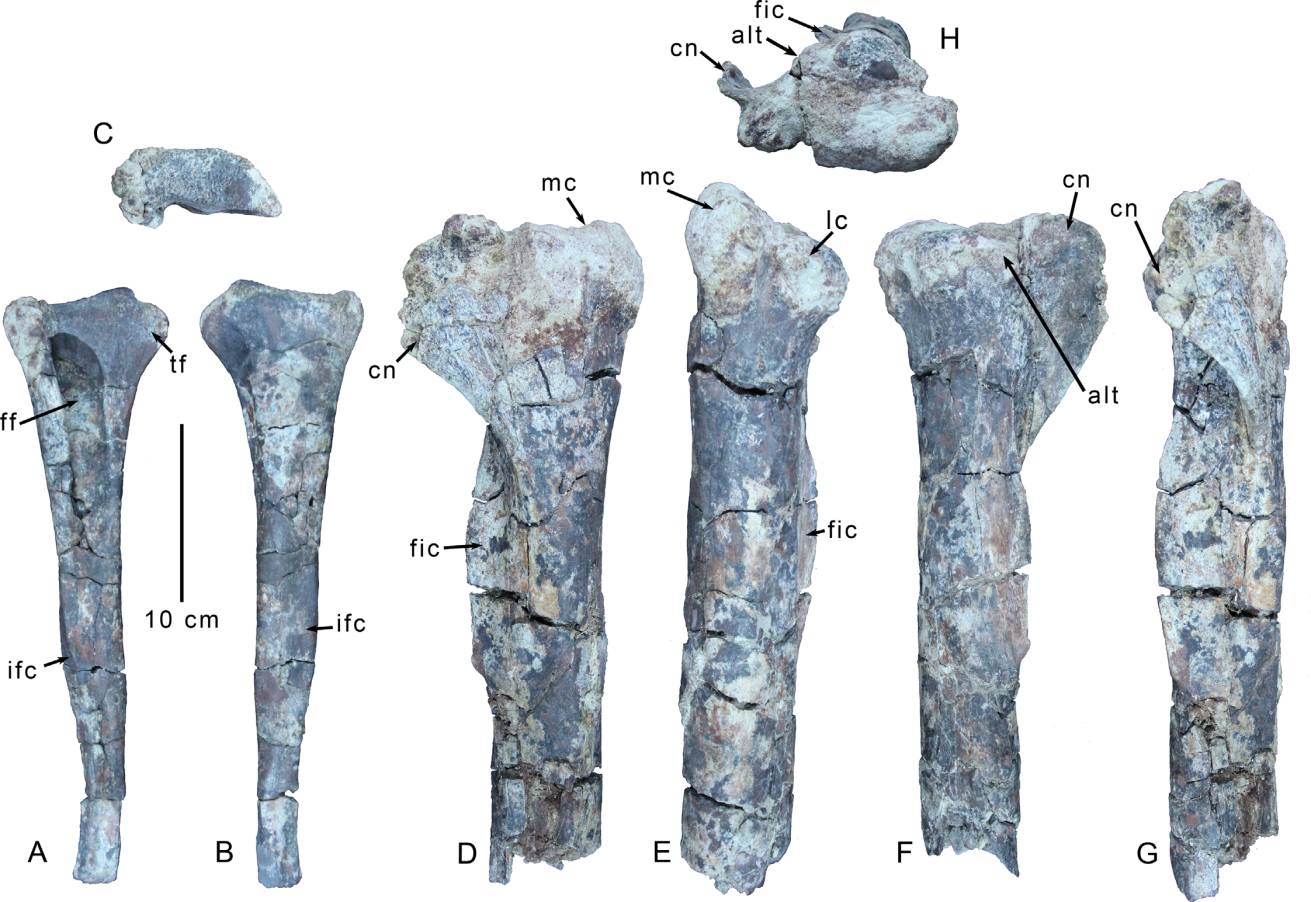
Right shank elements of *Gualicho shinyae*. Partial right fibula of the holotype specimen of *Gualicho shinyae* in (A) medial, (B) lateral, and (C) proximal views. Partial right tibia in (D) medial, (E) posterior, (F) lateral, (G) anterior, and (H) proximal views. Abbreviations: alt, anterolateral process; cn, cnemial crest; fic, fibular crest; ff, fibular fossa; ifc, crest for insertion of m. iliofibularis; lc, lateral condyle; mc, medial condyle; tf, triangular (posterior) flange.

The proximal part of a right tibia is preserved, broken approximately 14 cm distal to the end of the fibular crest (Fig 11). The proximal surface is heavily abraded and the medial surface of the cnemial crest is lost to erosion. The medial condyle is considerably more robust than the lateral condyle, and it projects proximally to a level above the proximal extents of both the lateral condyle and the cnemial crest (Fig 11E and 11F). The medial condyle also extends farther posteriorly than the lateral condyle (Fig 11H). Most of the proximal articular surface is rugose, but is also abraded and still has some matrix attached. A deep notch separates the medial and lateral condyles posteriorly, as is typical of tetanurans [7, 26]. Both condyles grade relatively smoothly into the tibial shaft distally, and do not form pronounced hoods or shelves. A low, but distinct (~2cm across) tuberosity projects into the incisura tibialis (Fig 11F), just anterior to the articular surface of the lateral condyle and may be homologous to the "anterolateral process" [38] (= "craniolateral process" and "ventral process"[53]). Benson et al. [38] noted a strongly ventrally curving anterolateral process of the lateral condyle in both *Australovenator* and *Neovenator*, but it is also present in *Deltadromeus* (SGM Din2). A very low ridge of bone extends anteromedially and slightly proximally from this tuberosity and runs across the lateral face of the cnemial crest. This low ridge divides the incisura tibialis (located distal to the ridge), from a smaller, weakly concave, triangular fossa proximal to the ridge. Just below the anterolateral tuberosity, the lateral edge of the tibial shaft extends down toward the fibular crest. The cnemial crest projects primarily anteriorly and does not rise very high proximally, barely clearing the proximal articular surface. It thus differs from the rectangular and strongly anterodorsally oriented cnemial process diagnostic of abelisauroids like *Majungasaurus* [8, 61], and also from the anterodorsally pointed cnemial crests of some allosauroids including *Sinraptor* [37] and *Giganotosaurus* (MUCP Pv CH 1). It exhibits a strong lateral curl to its anterior end (Fig 11G), such that its tip reaches the level of the anterolateral edge of the tibial shaft in anterior aspect. There is no evidence of a "posteroventral ridge" [53] on the lateral face of the apex of the cnemial crest, though the tip of the crest is broken. The medial face of the cnemial crest that still preserves the outermost cortical bone is covered by thick striations for soft tissue attachment. The crest extends distally and grades into the tibial shaft just below the level of the proximal end of the fibular crest.

The fibular crest does not project far lateral to the tibial shaft (Fig 11D–11F), but is proximodistally extensive. It grades smoothly into the tibial shaft proximally, but exhibits a more abrupt, tab-like distal border. Unlike a number of coelophysoid and ceratosaurian species, in which the fibular crest extends proximally to about the level of the lateral condyle [26], the fibular crest arises well below the lateral condyle in *Gualicho*. The edge of the fibular crest is slightly thickened and more rugose than the base of the crest. The posterior face is more heavily striated than the anterior one. The tibial shaft is an anterolaterally-posteromedially elongate ellipse in cross section. The anteromedial face of the tibial shaft is flattened, whereas the posterolateral face is rounded.

The proximal section of the right fibula is preserved, but most of the shaft distal to the m. iliofibularis tubercle is missing (Fig 11A–11C). Its proximal articular surface is an anteroposteriorly elongate ellipse, with a weak saddle-shaped concavity in the middle. A strong tab-like triangular flange projects posteriorly and slightly ventrally from the posterior edge of the proximal articular facet (Fig 11A). This flange is rounded and rugose proximally, and separated from the main articular facet by a shallow cleft. Distally, this flange grades smoothly into the posterior edge of the fibular shaft as a sharp ridge. The medial face of this flange bears a shallow sulcus that runs parallel to the much larger and more expansive medial fibular fossa. A similar sulcus is also present in *Giganotosaurus* (MUCP Pv CH 1). This shallow sulcus terminates distally before the ridge attenuates. Opposite this, the lateral side of the ridge is also marked by a proximodistally elongate fossa that is shallow and may be a site of muscle attachment.

The medial fibular fossa is extremely large and deep, and takes up almost the entire medial surface of the fibula, though it does not appear to invade any part of the robust posterior flange (Fig 11A). The medial fossa is deepest proximally, and grades out onto the medial shaft of the fibula slightly above the level of the m. iliofibularis tubercle. The proximal rim of the fossa is sharp and forms a hood over a portion of the fossa. Unlike non-tetanuran theropods such as *Syntarsus* [31], *Masiakasaurus* [55], and *Ceratosaurus* [60], there is no oblique ridge bordering the proximal rim of the medial fossa.

The m. iliofibularis tubercle is formed as an elongate, triangular flange (Fig 11A and 11B), as in *Deltadromeus* (SGM Din2), *Elaphrosaurus* (MB.R. unnumbered), and *Masiakasaurus* [55]. The rostral face of the flange bears a broad, shallow sulcus bordering the medial surface of the fibula. Distal to the m. iliofibularis tubercle, the shaft of the fibula is D-shaped in cross section and its lateral face is slightly convex, whereas its medial edge is flat.

**Fig 12:**
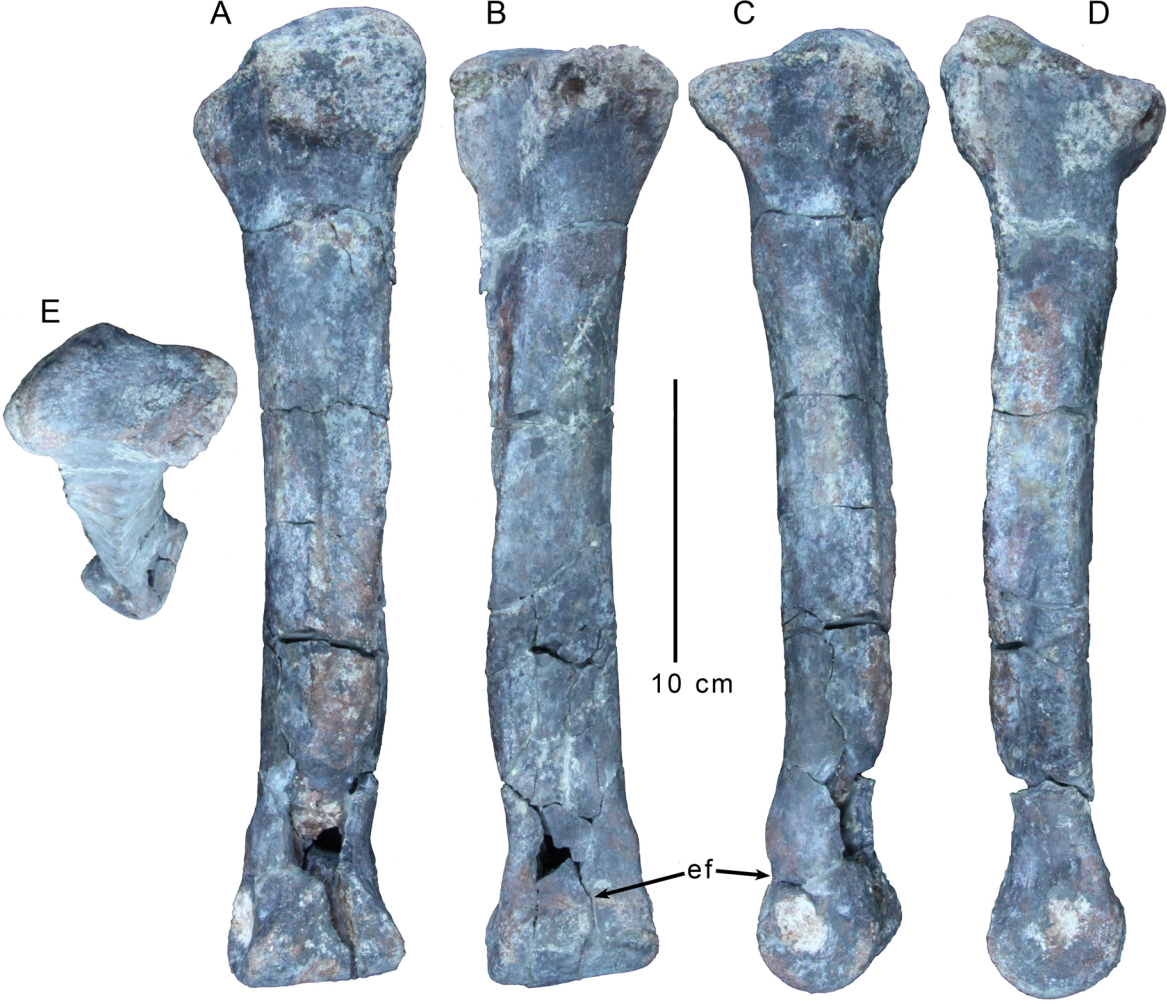
Left third metatarsal of *Gualicho shinyae*. Left third metatarsal of the holotype specimen of *Gualicho shinyae* in (A) posterior, (B) anterior, (C) medial, (D) lateral, and (E) proximal views. Abbreviation: ef, extensor fossa.

The left third metatarsal is almost complete (Fig 12). Its proximal articular surface is weakly concave, with raised anterior and posterior borders (Fig 12C and 12D). It is weakly figure 8-shaped in proximal view (Fig 12E), with slightly indented medial and lateral borders and convex rostral and caudal borders. The posterior edge of the proximal articulation is markedly wider than the anterior one and about as wide as the distal articulation, as seen in some ceratosaurs including *Elaphrosaurus* [29](MB.R. unnumbered) and *Majungasaurus* [61]. By contrast, most tetanurans have a third metatarsal with an "hourglass" shape in proximal aspect, with a wider anterior edge, a pinched middle section, and a posterior edge that is narrower than the anterior one [26], though *Acrocanthosaurus* is a notable exception to this pattern [35]. The raised anterior and posterior borders significantly overhang the shaft (Fig 12C and 12D), with the caudal edge bordering a massive, squared-off posterior process (Fig 12A), similar to ones observed in *Liliensternus* (MB R 2175), and especially *Elaphrosaurus* (MB.R. unnumbered). This block is extremely robust and rugose, and forms a distinct shelf that abruptly transitions to the metatarsal shaft, which is heavily marked by striations below it.

The robust metatarsal shaft is slightly bowed medially, an effect that is accentuated by a distal medial flange on the anterior surface (Fig 12B). Approximately seven to eight centimeters below the proximal end there is a low circular tuberosity on the anterior face of the metatarsal shaft. The medial articular surface for Metatarsal II is much more distinct than the lateral articular surface for Metatarsal IV, and the former extends as a broad concavity over almost the entire medial face of the shaft. The posteromedial edge of the metatarsal shaft forms a distinct ridge for insertion of the digital flexors (Fig 12A). A weaker ridge makes up the posterolateral edge of the metatarsal shaft, though it is not as extensive proximally. The posterior face of the shaft between these two ridges is mostly flat. The proximal end of the proximolateral ridge curls across the lateral face of the metatarsal shaft moving proximally and does not make contact with the proximal end of the metatarsal. The anteromedial edge of the metatarsal shaft, which marks the anterior border to the articular sulcus for metatarsal II, is not well developed proximally, but is better developed on the distal half of the metatarsal shaft and projects strongly medially as a distinct flange in anterior aspect. The flange grades smoothly back into the metatarsal shaft distally, just proximal to the expansion of the distal articular end of the metatarsal. A weak extensor fossa is present on the anterior face of the distal metatarsal (Fig 12E and 12F). It is bounded laterally and medially by two marked tuberosities, as in many theropods including *Allosaurus* [33], *Piatnitzkysaurus* (MACN CH 895), and *Torvosaurus* [62]. The lateral of these tuberosities is more robust and situated further proximally than the medial tuberosity. The distal end of the extensor fossa grades relatively smoothly onto the proximal end of the distal articular surface (i.e., the latter surface does not create a distinct proximal "shelf" connecting to the metatarsal shaft). Posteriorly, the transition from shaft to distal articular surface is slightly constricted.

The distal end of the metatarsal is transversely expanded, and the distal articulation is much broader than deep in distal aspect, a condition also observed in *Elaphrosaurus* (MB.R. unnumbered), *Majungasaurus* [61], and *Torvosaurus* [62] (FMNH PR 3060). The articular surface is smoothly rounded distally and lacks a distinct ginglymus and its exposure is triangular with a proximal apex (Fig 12A). Collateral ligament pits are well developed and appear to be relatively symmetrical in development, though both are still partially obscured by matrix.

**Fig 13:**
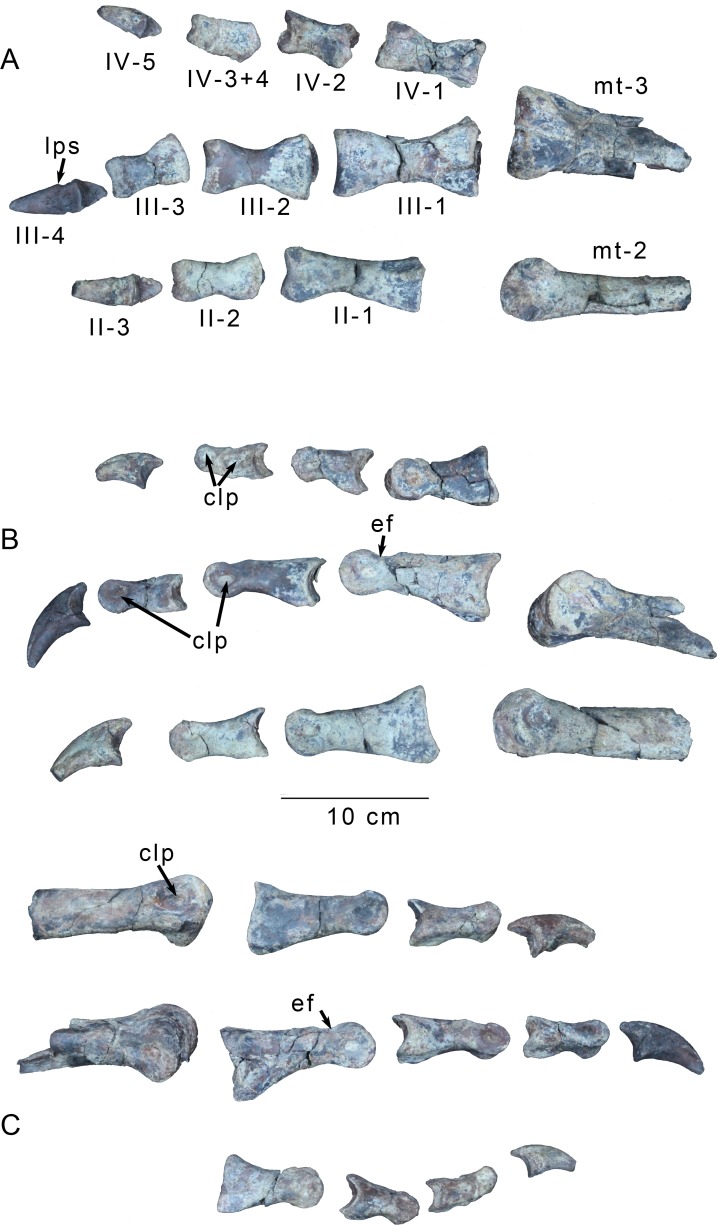
Right foot of *Gualicho shinyae*. Right pedal elements of the holotype of *Gualicho shinyae* including distal ends of metatarsals II and III and phalanges of digits II-IV in (A) ventral, (B) medial, and (C) lateral views. Combinations of Roman and Arabic numerals in (A) identify individual phalanges. Abbreviations: clp, collateral ligament pit; ef, extensor fossa; mt II, metatarsal II; mt III, metatarsal III.

The distalmost portion of the right metatarsal III is also preserved (Fig 13). It is slightly larger than the left element, particularly in its mediolateral breadth. The overall morphology is similar to the left element with several notable exceptions. The collateral ligament pits in the right element are much deeper and more distinctly rimmed. Also, the proximolateral tuberosity bounding the extensor fossa is not present in the right element, though the smaller distomedial tuberosity is present.

Only the distal end of the right metatarsal II is preserved (Fig 13). The shaft is quadrangular in cross section and deeper than wide. The anterior, medial, and posterior faces of the shaft are rounded, whereas the lateral border is flat throughout its preserved length. On the posterior face, a strong posterolateral edge that defines the posterior border of the flat articular surface for MT III is evident and is more pronounced along its distal half. There is no extensor fossa above the distal articular surface. The distal articulation is slightly asymmetrical and weakly canted medially. The anterior and distal portions of the distal articular surface are bulbous and undivided. The posterior hemicondylar rims are also asymmetrically developed on the distal surface (Fig 13A). The narrower medial hemicondyle extends further posteriorly and proximally than the lateral one, though the posteriormost tip of the lateral hemicondyle is broken off. The posterior end of the medial hemicondyle is everted slightly medially. A wide sulcus separates the hemicondyles in plantar view. The collateral ligament pits are asymmetrically developed. Both are deep and bear distinct rims, but the lateral pit is distinctly longer proximodistally and more teardrop-shaped, whereas the medial pit is largely circular.

All phalanges of the three principal digits of the right foot are preserved, though the tips of unguals II-3 and IV-5 are broken (Fig 13). All preserved non-terminal phalanges with the exceptions of IV-3 and IV-4 are elongate and slender, with their shafts constricted between the expanded articular ends in dorsal view, unlike the short and stout phalanges of carcharodontosaurids [2, 35], and abelisaurids such as *Majungasaurus* [61]. They also exhibit well-defined collateral ligament pits and extensor pits just proximal to the dorsal ends of the distal articulations (Fig 13B and 13C). The unguals are short, curved and triangular rather than elliptical in cross section, and the ungual of pedal digit II is symmetrical unlike those of abelisauroids [8]. Very weak flexor tubercles are present, but the claw sheath grooves are not caudally forked as in abelisauroids [63]. The grooves are well defined and the proximal portion of their ventromedial and ventrolateral edges form little spurs on the ventral aspect of the ungual (Fig 13A) as in *Beishanlong* [64].

### Phylogenetic results

**Fig 14:**
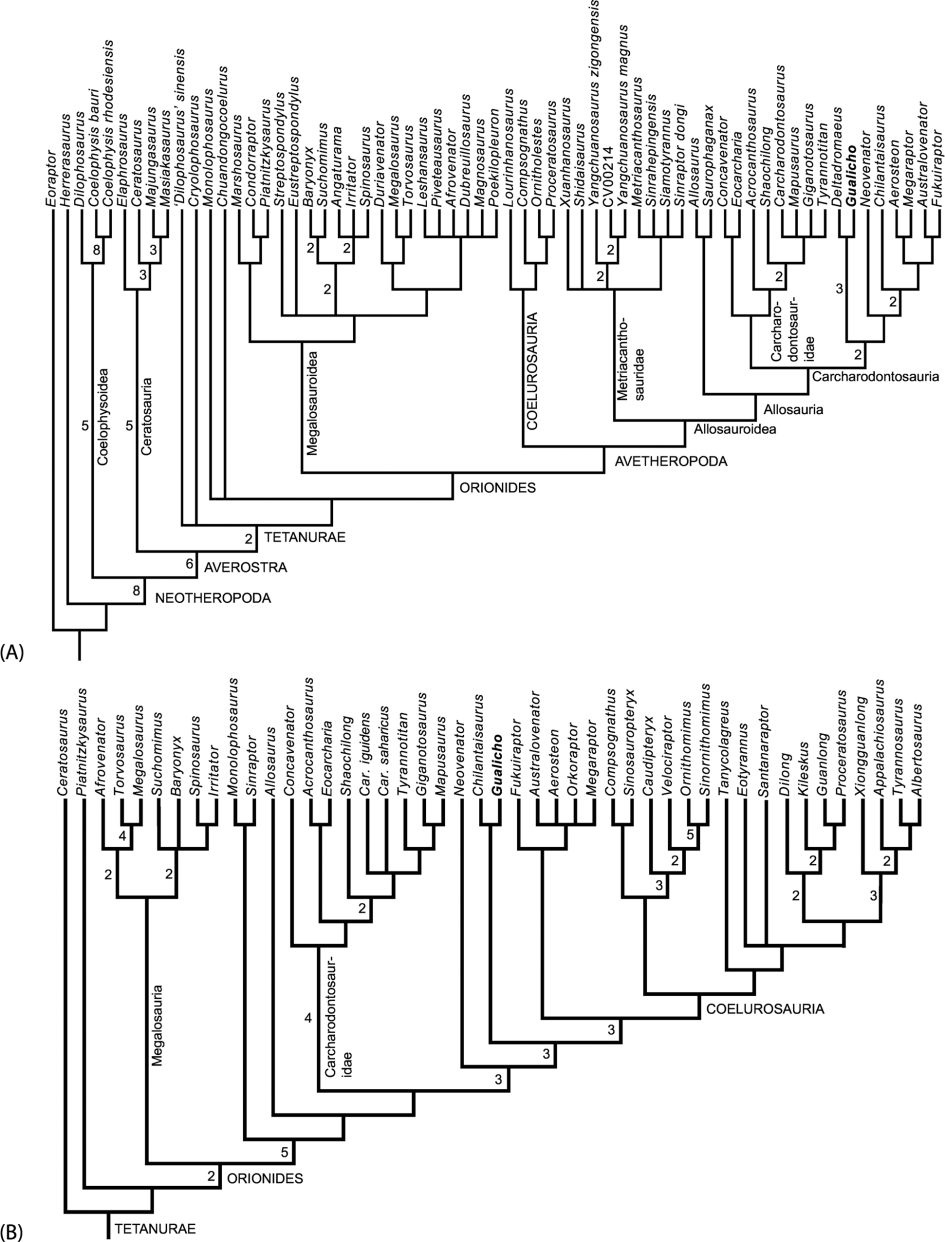
Strict consensus trees of theropod relationships showing alternative phylogenetic positions for *Gualicho*. (A) Strict consensus of 972 Most Parsimonious Trees (MPTs) of 1075 steps each resulting from analysis of the modified Carrano et al. [7] dataset with *Gualicho* and *Deltadromeus* added. Major clade names follow usage in [7]. Numbers above nodes reflect branch support values in excess of 1. (B) Strict consensus of four MPTs of 945 steps each resulting from the analysis of the modified Porfiri et al. [11] dataset with all characters treated as unordered. Clade names and branch supports as in (A).

Addition of *Gualicho* and *Deltadromeus* to the Carrano et al. [7] character-taxon matrix resolves them as basal carcharodontosaurians and sister to the neovenatorid radiation (Fig 14A). This result is recovered whether the nine new characters are included or not. Whereas the phylogenetic position of these two fragmentary specimens appears well resolved, support is relatively low, with Bremer support [65] values of 1 for most nodes along the spine of the tetanuran radiation. However, the sister-taxon relationship between *Gualicho* and *Deltadromeus* is relatively robust (branch support = 3), despite the fact that both specimens are incomplete. Addition of *Gualicho* introduces some character conflict, with tree length increasing from 1044 to 1063 steps, and addition of both *Deltadromeus* and *Gualicho* increases tree length to 1075 steps. Comparison to the tree length increases associated with inclusion of other taxa as calculated with a modified version (see S6 File) of the ‘Term_lengths’ script (http://phylo.wdfiles.com/local—files/tntwiki/Term_lengths.run) suggests that the tree length increments associated with *Gualicho* (9 steps) and *Deltadromeus* (10 steps) are well below increments associated with other taxa such as *Dilophosaurus* (29 steps), *Ceratosaurus* (19 steps), and *Majungasaurus* (25 steps). The conflict is borne in characters for which *Gualicho*, and more specifically, *Deltadromeus*, exhibit character states that are either plesiomorphic for tetanurans or shared with Ceratosauria. Indeed, if *Gualicho* is excluded from the analysis, *Deltadromeus* groups with ceratosaurs, mirroring previously published results [8, 30]. Constraining these two taxa to both be ceratosaurs results in an increase in tree length to 1079 steps.

In the analysis of the modified Porfiri et al. [11] dataset, *Gualicho* was recovered near the base of Coelurosauria (Fig 14B), well removed from either didactyl tyrannosaurids, or the megaraptoran clade it was close to in the Carrano et al. [7] dataset. Surprisingly, however, our reanalysis following changes to the matrix found *Neovenator* as sister taxon to a clade of coelurosaurs plus *Gualicho* and megaraptorans rather than as a member of Allosauroidea. We did not find support for megaraptorans as members of Tyrannosauroidea as previously reported [11, 12] after rescoring a number of characters in those analyses (see S1 Text) and running all traits as unordered, although megaraptorans were found to be closer to tyrannosauroids than to the included carcharodontosaurids. The very different results of these two analyses are predicated on significant differences in both taxon- and character sampling, and only a more comprehensive analysis beyond the scope of this description can resolve the disagreement.

### Multivariate analyses results

#### Principal Coordinates results

**Fig 15:**
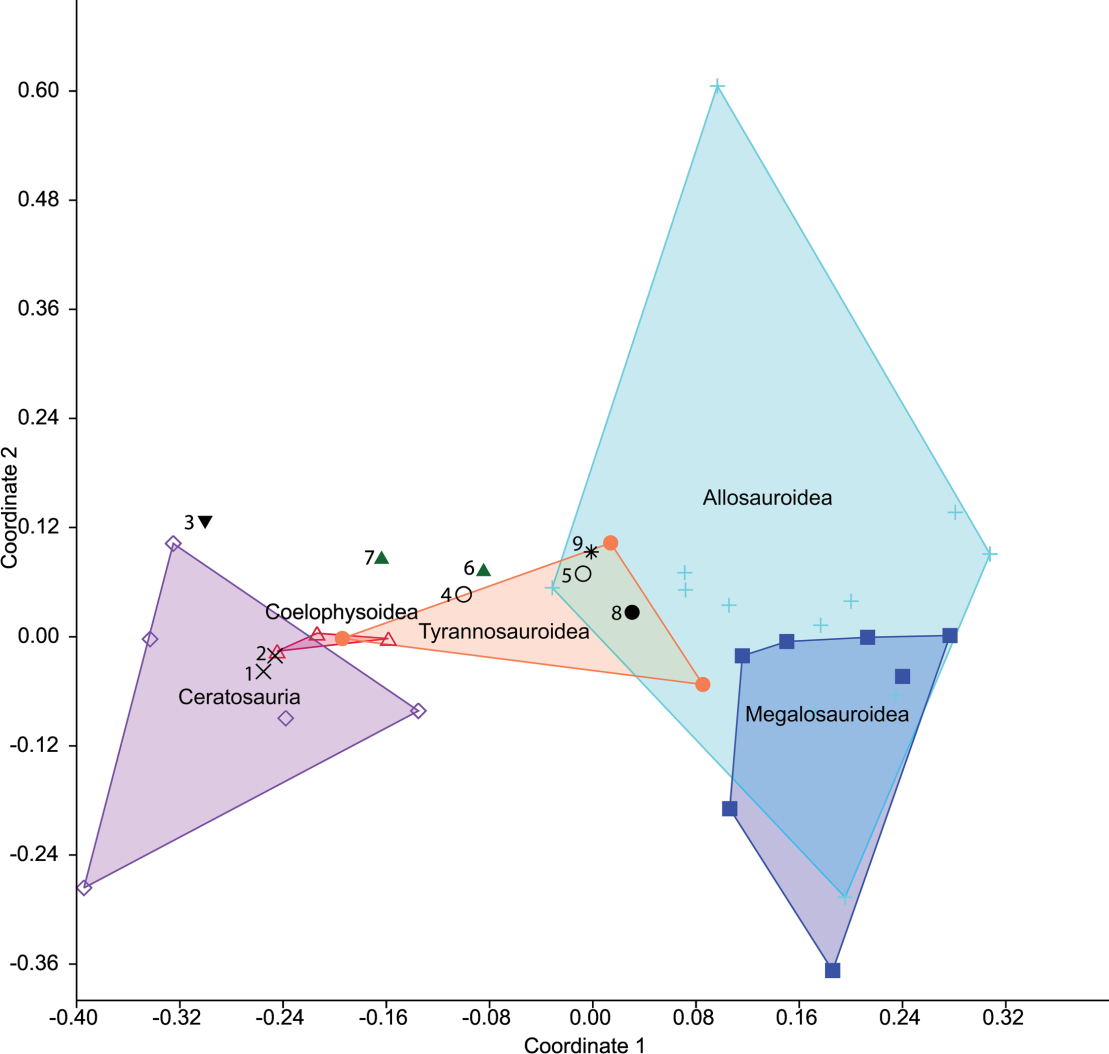
Principal coordinates morphospace for forelimb traits in select non-avian Theropoda. PO1 versus PO2 morphospace based on Gower metric for 33 binary forelimb characters in a broad taxonomic sample of non-avian theropods. Datapoints marked with numerals represent the following taxa: 1, *Eoraptor*; 2, *Herrerasaurus*; 3, *Deltadromeus*; 4, *Compsognathus*; 5, *Ornitholestes*; 6, *Harpymimus*; 7, *Struthiomimus*; 8, *“Dilophosaurus” sinensis*; 9 *Gualicho*. Convex hulls correspond to groups in identified in Fig 14A.

The morphospace distribution of taxa along the first two Principal Coordinate (PO) axes is shown in Fig 15. A broad phylogenetic trend is discernible with outgroup taxa *Herrerasaurus* and *Eoraptor* and basal clades such as coelophysoids and Ceratosauria skewed toward the left, and most tetanurans clustering further to the right in morphospace. Coelurosaurian taxa generally considered to have reduced or weaker forelimbs, such as tyrannosaurids, and ornithomimids are skewed toward the left part of morphospace (negative PO1 values), however. Allosauroids and megalosauroids are concentrated toward the right, with basal tetanurans and basal coelurosaurs closer to the 0,0 origin. *Gualicho* is close to the 0,0 origin, and overlaps with allosauroids and the basal coelurosaur part of morphospace, but is relatively far from taxa/ clades exhibiting a foreshortened or reduced forelimb morphology. The basal coelurosaurs *Ornitholestes* and the tyarnnosauroid *Tanycolagreus* are the closest taxa in morphospace, followed by the basal tetanurans ‘*Dilophosaurus’ sinensis*, *Szechuanosaurus* (= *Yangchuanosaurus* in [7]), and CV00214.

#### Principal Components results

**Fig 16:**
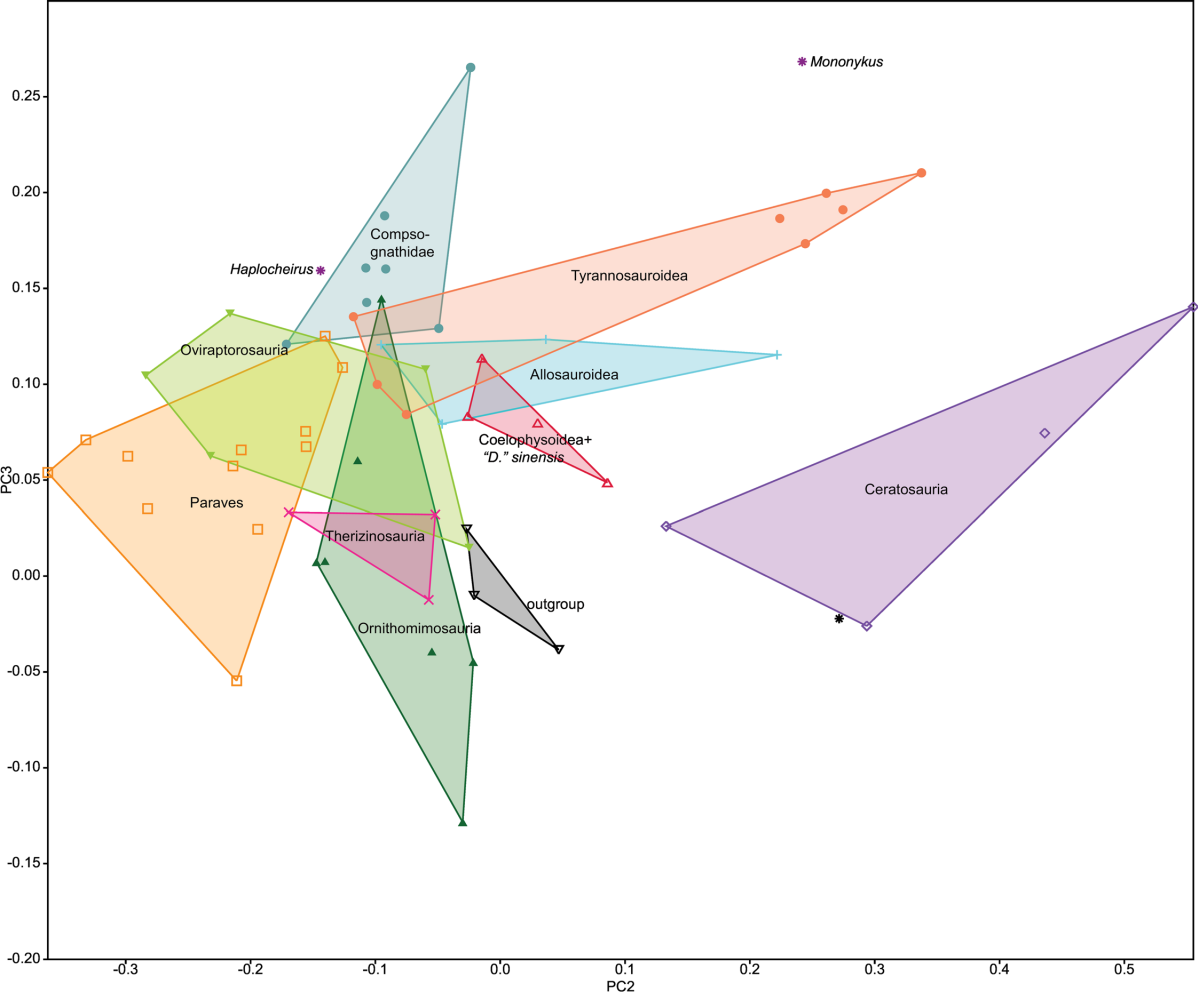
Forelimb morphospace for select theropods. Morphospace (unstandardized pPC2 versus pPC3 scores) of three forelimb measurements and femoral length in select non-avian theropod taxa analyzed under the Lambda model. Convex hull names follow Fig 14A and [21]. Almost identical patterns are found when data are analyzed under the Brownian Motion model.

Our pPCA analyses indicate a strong degree of phylogenetic influence (Lambda = 0.8880) on pPCA scores, so we will focus on the results of the analysis conducted under the Lambda model. (Fig 16). All four limb measurement variables show strong negative loadings between -0.9125 and -0.9697 on the first pPC axis indicating that size differences explain the bulk of the variance in this parameter, as also noted by [17]. Positive loadings are found along pPC2 (Brownian Motion/ Lambda values) for the humerus (0.0373/0.0474) and femur (0.3124/0.2771), while the length of metacarpal I is negatively loaded (-0.2137/-0.4004). The radius has a positive loading when the Brownian Motion model is employed (0.0703), but ha a negative loading under the Lambda model (-0.10214330). For pPC3, both the humerus (-0.0576/-0.0778) and radius (-0.2224/-0.1515) are negatively loaded, whereas metacarpal I (0.0803/0.1328) and femur (0.1514/0.0680) receive positive loadings. In pPCA morphospace (Fig 16), outgroup taxa such as *Eoraptor*, and non-tetanuran theropods including coelophysoids, and ‘*Dilophosaurus*’ *sinensis* fall close to the 0,0 origin. Long-limbed maniraptoran taxa mostly occupy the left quadrants of the mophospace, and overlap to a small degree with compsognathids, ornithomimosaurs, and basal tyrannosauroids that have negative pPC2 scores, but positive pPC3 scores. Non-avian theropods with abbreviated forelimbs including tyrannosaurids, the alvarezsaurid *Mononykus*, and abelisaurids are mainly distributed in the upper right quadrant of morphospace. *Gualicho* is found adjacent to the ceratosaurian morphospace, with pPCA scores most similar to those of the basal ceratosaurs *Limusaurus* and *Eoabelisaurus* among the sampled taxa under both model implementations (Fig 16).

All clades with abbreviated forelimbs exhibit a consistent pattern in which basal members occur to the left of more derived, shorter armed relatives in pPC2/ pPC3 morphospace. Interestingly, for alvarezsauroids and tyrannosauroids, the basalmost members (*Haplocheirus*, *Guanlong*) map close to other basal coelurosaurs. The derived coelurosaurian clades Ornithomimosauria, Therizinosauria and Oviraptorosauria, which were likely herbivorous [66], overlap in morphospace to the right of Paraves and below the compsognathid convex hull.

The typical PCA yields generally similar results to the phylogentic PCAs (though with reversed loading polarities), but most grades and clades exhibit a greater degree of overlap with the exception of Ceratosauria, which occupy their own unique quadrant (S2 Fig), and the parallel trends among short limbed taxa of evolving toward the same quadrant are less pronounced though still evident. In the typical PCA result *Gualicho* falls just within the convex hull for Ceratosauria, again close to *Limusaurus* and *Eoabelisaurus*. Both the pPCA and PCA plots suggest that the forelimb of *Gualicho* is proportionately reduced in a manner similar to those of abelisauroid ceratosaurs more derived than *Eoabelisaurus*, despite marked differences in manus anatomy.

## Discussion

*Gualicho shinyae* represents a new tetanuran theropod taxon from the Huincul Formation, which is distinct from other coeval and sympatric theropods lineages such as Carcharodontosauridae represented by *Mapusaurus* [2], and Abelisauridae represented by *Ilokolesia* [4] and *Skorpiovenator* [5], thus adding to the known taxonomic and phylogenetic diversity of Neuquén Group dinosaurs. It exhibits a peculiar diagnostic character combination including traits that converge on derived character states observed in various remotely related clades including ceratosaurs (metatarsal III proximal dimensions [8]), tyrannosaurids (reduced forelimb with didactyl manus [67]), and megaraptorans (elongate dorsal centra, hooked process on lateral tibial condyle [38]). This mosaic of synapomorphies from multiple, distantly related groups renders our understanding of the affinities of *Gualicho* highly uncertain. As illustrated by the two phylogenetic analyses conducted here on datasets that overlap insufficiently in character, and especially, taxon sampling, *Gualicho* occupies different derived positions within Tetanurae, depending on which matrix is used. A more robust evaluation of its affinities requires a more comprehensive review of theropod relationships that samples widely among ceratosaurians, allosauroids, and basal coelurosaurs, but which is beyond the scope of this study.

At lower taxonomic levels, *Gualicho* shares several derived characters with the African theropod *Deltadromeus* from the nearly coeval Kem Kem beds of Niger, including a scapula with a sinuous border on the transition from the acromion process to the blade, reduced distal humeral articulations, and an expanded lobe bearing a medial trough on the proximocaudal aspect of the fibula. Both taxa also share a proximocaudally expanded articulation on Metatarsal III, a trait also seen in ceratosaurs (antarctometatarsal condition of Carrano and Sampson [8]). A sister taxon relationship between *Deltadromeus* and *Gualicho* is supported in our analysis of tetanuran relationships. Furthermore, with a Bremer Support value of 3, this clade is among the most well supported relationships in the tree. Nevertheless, notable differences exist between the two taxa, such as in the anatomy of the humerus, which is longer than the scapula in *Deltadromeus* (unlike the original reconstruction [9]), but much shorter in *Gualicho*. Moreover, *Gualicho* exhibits a mediolaterally expanded humeral head and moderately well-developed muscle attachment including a lobate deltopectoral crest set at a right angle to the expanded and pointed internal tuberosity. By contrast, the humeral head is hemispherical in *Deltadromeus*, and the internal tuberosity and deltopectoral crest are reduced, traits otherwise observed and considered to be synapomophies of ceratosaurian theropods [8, 26], or independently acquired within subclades thereof such as Abelisauridae and Noasauridae [20]. Unlike ceratosaurs, however, *Deltadromeus* does exhibit a lobate, albeit very small, deltopectoral crest as in *Gualicho* and most allosauroids.

The affinities of *Deltadromeus* have long been uncertain. It was originally described as a basal coelurosaur [9], but was subsequently interpreted as a basal ceratosaurian [8, 30]. In this regard, it is interesting to note that *Gualicho* does exhibit some apparent ceratosaurian synapomorphies, most notably a robust MT III with posteriorly expanded proximal articulation [8, 26] and a flange-like m. iliofibularis tubercle [26], but has a distinctly tetanuran manus morphology [7], and carcharodontosaurian traits such as mid-dorsal centra with slit-like pneumatic foramina [7, 38]. In our analysis, the unconstricted metatarsal condition and fibular flange morphology are interpreted as a synapomorphy of *Gualicho* and *Deltadromeus*, acquired convergently with ceratosaurs. In spite of this apparent mosaic of ceratosaurian and tetanuran synapomorphies in these two atypical Gondwanan taxa, it is interesting to note their inclusion does not add significantly to tree length when compared to other species included in the phylogenetic analysis when accounting for completeness.

A number of characters shared by *Gualicho* and *Deltadromeus* relate to forelimb reduction in the broad sense (i.e., reduction in either length or robustness, or both), and are convergently present in other theropod clades. For example, ornithomimids (e.g., *Ornithomimus* RTMP 95.110.1) exhibit a slender, straight humerus with a sub-spherical head [26], a reduced deltopectoral crest [26, 64], and an unexpanded distal end with a shallow flexor fossa. All of these traits are observed in *Deltadromeus*, and the straight shaft and poorly developed distal articulation are also present in *Gualicho*, and some combination of these states are also observed in many flightless birds, for example *Dromaius* [68]. Although this set of traits commonly seen in reduced and vestigial forelimbs of theropods raises the possibility that potential synapomorphies in the distal humerus uniting *Gualicho* and *Deltadromeus* could be homoplastic, other synapomorphies are distributed throughout the pectoral girdle and hindlimb, arguing against this grouping being the result of a single anatomical region undergoing parallel reduction.

Another trait related to forelimb reduction is the apparent didactyly of the manus of *Gualicho*, which resembles those of tyrannosaurids in having the third digit reduced to a metacarpal splint. Closer scrutiny, however, reveals a number of detailed differences in manual anatomy. In tyrannosaurids, the carpal elements are reduced and proximodistally flattened [67], whereas in *Gualicho* the semilunate and scapholunare carpals retain a more complex shape typical of the carpal elements of most non-coelurosaurian tetanurans [69]. Furthermore, the manus of *Gualicho* differs from tyrannosaurids [67] in having a proportionately more robust metacarpal I with a rectangular, rather than triangular, proximal articulation in end view.

The PCO analysis is consistent with the interpretation of our phylogenetic result implying that forelimb reduction in *Gualicho* likely occurred independent of other lineages with reduced forelimbs such as abelisauroids, alvarezsaurids, ornithomimosaurs, and especially didactyl tyrannosaurids. Interestingly, *Gualicho* falls close to the abelisauroid morphospace in the pPCA analysis and within the convex hull for abelisauroids in PCA morphospace, despite the fact that the manus of *Gualicho* differs substantially in anatomy from that of abelisauroids and that this result is inconsistent with our phylogenetic results, even though *Gualicho* (and *Deltadromeus*) share some derived hindlimb traits with ceratosaurs. The differing results of the PCO and pPCA /PCA analyses suggest that while forelimb reduction in *Gualicho* was not accompanied by the same suite of discrete or qualitative traits observed in ceratosaurs, reduction in scaling of elements may have followed similar patterns.

Both multivariate analyses suggest parallel evolutionary trends in taxa with abbreviated forelimbs. In the PCO plot clades such as tyrannosaurids and abelisaurids with shortened forelimbs occur far to the left along PCO1 and even within these clades, more derived members occur to the left of more basal relatives. However, some noteworthy differences also emerge from comparisons of these results. In the PCO plot ornithomimosaurs exhibit a trend parallel to that of tyrannosaurids whereas in the pPCA plot they exhibit nearly orthogonal trajectories indicating that patterns of trait change and changes in forelimb proportions are not always correlated. Likewise, *Gualicho*, which exhibits forelimb proportions similar to those of ceratosaurs, is more similar to most tetanurans, and basal coleurosaurs in particular in terms of the forelimb trait scorings sampled in the PCO analysis.

These results support classical "many-to-one" mapping models of convergence [70] in that there are many different ways to evolve a shortened or reduced forelimb across theropods. An important distinction, however, is that function likely differed across all these taxa with reduced forelimbs. Nevertheless, reduction of the forelimb in *Gualicho* exhibits some general commonalities with other tetanurans such as loss of digit III phalanges and reduction of its metacarpal. Though most similar to the tyrannosaurid condition, progressive reduction of digit III is seen in other tetanurans including *Megaraptor* (Digit III very slender), derived alvarezsaurids (loss of all Digit III elements and Digit II phalanges in *Linhenykus* [71]), *Caudipteryx* (loss of terminal phalanges of Digit III), and Ornithuromorpha (loss of all Digit III phalanges). *Gualicho* appears to be the phylogenetically basalmost, though not the temporally oldest, taxon to exhibit this developmental reduction pattern that is common among tetanurans.

A sister taxon relationship between *Gualicho* and *Deltadromeus* is consistent with other known global-scale faunal distributions, as many of the dinosaurs found in the Huincul Formation are closely related to species found in mid-Cretaceous strata across North Africa including the Kem Kem Compound Assemblage (KKCA) of Morocco [72], the equivalent Bahariya Formation of Egypt, and Echkar and Elhraz Formations of Niger [73]. While faunal differences exist between these poorly age-constrained units in North Africa, those may be related to poor sampling [73], so we will consider these assemblages as roughly equivalent for the purposes of this broad scale discussion. The Huincul Formation and KKCA represent among the latest occurrences of rebbachisaurid sauropods in the global record [25, 74] and the Huincul, Bahariya, and Echkar Formations yield latest definitive Gondwanan skeletal occurrences of carcharodontosaurids [7,75]. The Bahariya and Huincul Formations also share the earliest appearance of gigantic (>25 m) titanosaurian sauropods [1,76], which are also known from the roughly coeval Bayo Overo Member of the Cerro Barcino Formation, central Patagonia, and from the Bajo Barreal Formation of southern Patagonia [77]. The first appearance of abelisaurids with rugose facial ornamentation in South America is in the Candeleros [78] and overlying Huincul Formation [4,5], as well as in the Bajo Barreal Formation [79]. This is temporally equivalent to the first appearance of this clade in Africa, represented by the Cenomanian taxon *Rugops* from the Echkar Formation of Niger [30].

These strong faunal resemblances between strata in the Neuquén and San Jorge Basins of Patagonia and North African Cenomanian beds are intriguing, but difficult to interpret due to a lack of well sampled, age equivalent strata elsewhere [80]. Although these distributions provided evidence of Gondwanan-Laurasian vicariance during the Aptian to Santonian in a recent global analysis of Mesozoic biogeography [81], that analysis excluded all Gondwanan landmasses except for South America and Africa for mid-Cretaceous time slices, and is therefore not informative with respect to biogeographic patterns across Gondwanan continents. While data from Crocodyliformes suggests closer potential affinities between South America and Indo-Madgascar [82] than with Africa during the mid-Cretaceous, it is critical to bear in mind that sampling can have significant impact on any recovered biogeographic signal [83], and authors have noted that sampling is likely insufficient to adequately test biogeographic hypotheses for either dinosaurs [8] or crocodyliformes [84] in Gondwana. In this regard, patterns of local extirpation and a poor global ‘mid’-Cretaceous record have been invoked as critical factors in generating observed distribution patterns among theropods [7, 8], dinosaurs [85] and tetrapods in general [80], and better sampling is required before a robust explanation can be proffered for the similarities we note between the Huincul Formation and approximately age-equivalent strata in Africa.

Important differences between the coeval Huincul Formation (Neuquén Basin) and Bahariya Formation (Bahariya Oasis) faunas can be related to environmental differences. The KKCA and Bahariya Formation represent nearshore, mesic units [72, 76, 86, 87] characterized by a rich aquatic fauna (e.g., elasmobranchs, osteichthyes [88], crabs [89], semi-aquatic theropods like *Spinosaurus* [86]), whereas the Huincul Formation was deposited in a foreland basin far from the coast, as demonstrated by the absence of a rich aquatic fauna, and no evidence of northern Gondwanan spinosaurids in its otherwise rich dinosaur fauna. However, this paleoenvironment-based interpretation may be tempered by the presence of the Central Gondwanan Desert, which has been interpreted as a barrier for both spinosaurid dinosaurs and podocnemidoid turtles [75].

## Supporting Information

S1 Fig Photographs of the discovery and initial excavation of the holotype of *Gualicho shinyae*. (Upper left) Akiko Shinya next to parts of pubis of the holotype immediately after her discovery of the specimen. (Upper right) Initial excavation of specimen. (Lower left) Articulated right foot of the holotype of *Gualicho shinyae* during excavation. (Lower right) Authors Apesteguía (left), Makovicky (center), and Smith (right, behind Makovicky) at excavation site.
(TIF)

S2 Fig Results of traditional PCA on log-transformed data from S7. (TIF)

S1 File Taxon-character matrix modified from [7] with added characters and taxa. Matrix in TNT format modified from [7] with addition of *Gualicho* and *Deltadromeus* and nine new characters.
(TNT)

S2 File Taxon-character matrix modified from [11] with added characters and taxa. Matrix in TNT format modified from [11] with addition of *Gualicho* and four new characters.
(TNT)

S3 File Phylogenetic tree used for pPCA analysis of theropod forelimb reduction patterns. Nexus formatted tree file detailing relationships between the 60 taxa in the pPCA analyses.
(TRE)

S4 File Age ranges used to calibrate S3 File for pPCA. Text file with stratigraphic ranges for theropod taxa included in the pPCA analysis for calibrating the S3 File using STRAP [22].
(TXT)

S5 File Script for conducting pPCA analysis using files S8-S10. (TXT)

S6 File TNT script for determining increase in tree length with addition of each taxon in a data set. Script containing TNT macro to be run after dataset is processed.
(TXT)

S1 Table Data matrix used in PCO analysis of theropod forelimbs. (XLSX)

S2 Table Data matrix of forelimb and femoral measurements used in pPCA/PCA analysis of theropod forelimb reduction patterns. (XLSX)

S3 Table Log-transformed measurements for forelimb elements and femur used in pPCA analyses. Table with log-transformed data formatted as text for pPCA analyses in R (see S5 File).
(TXT)

S1 Text Decription of characters added and scorings changed in the two phylogenetic analyses. Description of new characters added to the phylogenetic analyses based on [7] and [11], and discussion and justification for re-scoring select character states in those data matrices.
(DOCX)

S2 Text Forelimb characters employed in PCO analysis. List of forelimb characters taken from [7] with two new characters added and analyzed with Principal Coordinates analysis.
(DOCX)
