# Supplementary figures and images for: An Unusual New Theropod with a Didactyl Manus from the Upper Cretaceous of Patagonia, Argentina
Source: PLoS One. 2016 Jul 13;11(7):e0157793. doi: 10.1371/journal.pone.0157793 (PMC4943716; doi:10.1371/journal.pone.0157793)

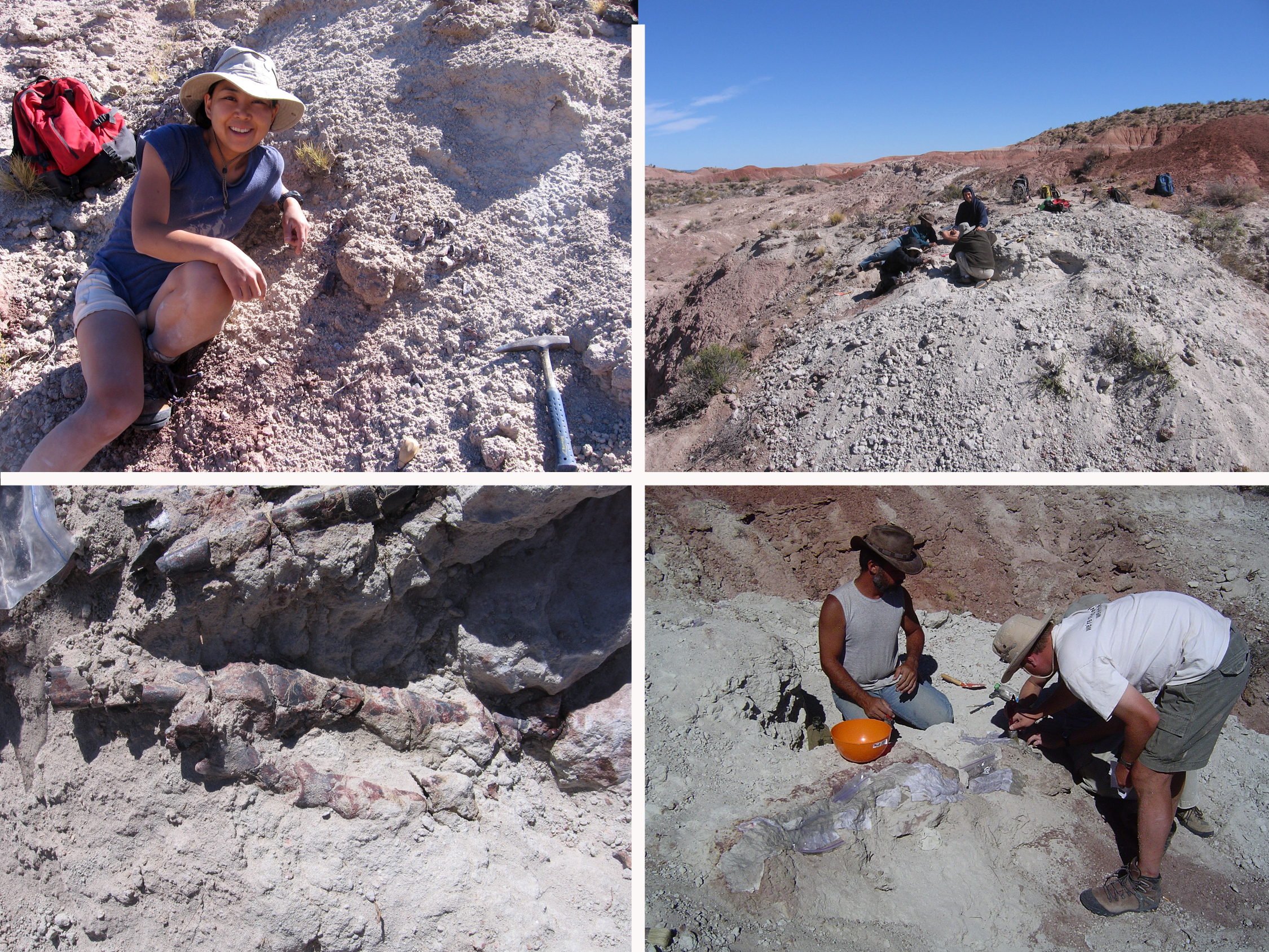

Supplement: S1 Fig — (Upper left) Akiko Shinya next to parts of pubis of the holotype immediately after her discovery of the specimen. (Upper right) Initial excavation of specimen. (Lower left) Articulated right foot of the holotype of Gualicho shinyae during excavation. (Lower right) Authors Apesteguía (left), Makovicky (center), and Smith (right, behind Makovicky) at excavation site. (TIF) [file pone.0157793.s001.tif]

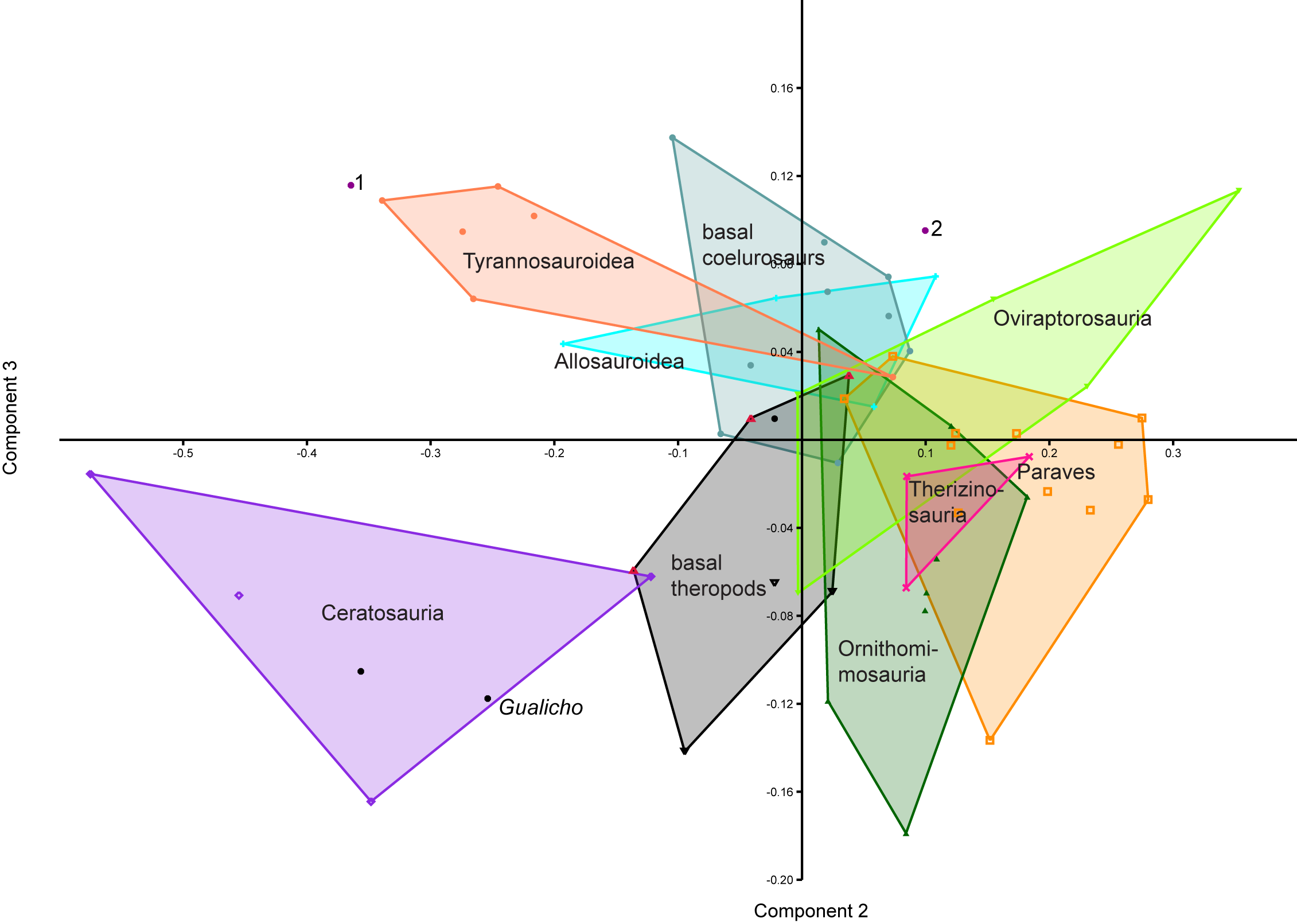

Supplement: S2 Fig — (TIF) [file pone.0157793.s002.tif]
