# Supplementary material for: An Unusual New Theropod with a Didactyl Manus from the Upper Cretaceous of Patagonia, Argentina
Source: PLoS One. 2016 Jul 13;11(7):e0157793. doi: 10.1371/journal.pone.0157793 (PMC4943716; doi:10.1371/journal.pone.0157793)
Supplement: S1 Text — Description of new characters added to the phylogenetic analyses based on [7] and [11], and discussion and justification for re-scoring select character states in those data matrices. (DOCX) [file pone.0157793.s012.docx]

S2: Supplementary Information to Phylogenetic Analyses

CHARACTERS ADDED TO CARRANO ET AL (2012) MATRIX:

352) Deltopectoral crest, anterior edge: straight or gently arced (0), or lobate (1).

353) Presacral vertebrae, pneumaticity of centrum: present in cervicals only (0), or present in cervicals and trunk vertebrae (1)

354) Pedal unguals, ventrolateral and ventromedial edges: without spurs (0) or with small spurs marking ends of crease between ventral and lateral/ medial edges (1)

355) Cervical and anterior trunk vertebrae, articulations: amphi- or platycoelous (0), or ball and socket articulation (1)

356) MTIII, proximal end: caudal rim as wide or narrower than rostral face (0), or caudal edge wider (1)

357) Fibula, medial face without proximal ridge (0) or proximal ridge present (1).

358) Scapula, acromion-blade transition: shaped as concave curve (0), or as a sinusoidal curve (1)

359) Flexor tubercles on pedal unguals: absent (0) or present (1)

360) Humeral shaft, lateral furrow on proximal half: absent (0) or present (1)

CHANGES TO CODINGS TO CARRANO ET AL (2012) MATRIX:

Character 320: Altered scoring to State 1 based on observation of UMNH VP 5278. We interpret this character as whether the incisura tibialis defines a notch between the lateral condyle and the base of the cnemial crest or not.

Character 341: We altered the coding in *Acrocanthosaurus* to State 0 from State 2, as the proximal end of the metatarsal II appears elliptical in proximal view. This is seen both in Currie & Carpenter (2000) and in photos of OMNH type and paratype material. In *Megrapator*, MT III is incomplete and the original coding appears to have been scored based on the morphology of the proximal end of MT IV. However, it seems more prudent to score this as 1/2 as the size of the notch may be hard to infer from MT IV anatomy alone.

CHARACTERS ADDED TO PORFIRI ET AL (2014) MATRIX:

285) Quadrate shaft, pneumatic foramen on posterior face: absent (0) or present (1)

286) Premaxillary symphysis, ventral view: acute, V-shaped (0) or broad, U-shaped (1)

287) MC II, proximal end: narrow, less than twice as wide as midshaft (0) or expanded, twice as wide as midshaft (1)

288) Manus: more than two digits (0) or only tow digits present (1)

CHANGES TO CODINGS IN PORFIRI ET AL (2013):

Character 4: Porfiri et al (2014) describe the isolated premaxillary tooth of *Megaraptor* as being D -shaped, but this is incorrect. While, the mesial and distal carinae are set at about 180 degrees apically, the mesial carina then twists lingually as it approached the base as and is roughly perpendicular to the distal carina near the root (Porfiri et al. 2014, fig. 2), as in many other theropods including *Allosaurus* and *Dromaeosaurus*,. The tooth does not have the classical U shape of a tyrannosaurid premaxillary tooth (see Hendrickx & Mateus 2014), but is rather J shaped at the base. The same applies to *Fukuiraptor*

Character 5: The apex of the premaxillary tooth is clearly positioned closer to the distal carina (Porfiri et al. 2013, fig. 2), not between the carinae as is characteristic of the 'straight' condition

Character 8: In *Acrocanthosaurus*, both maxillary and especially dentary crowns appear to have an apex that is projected beyond the base of the distal carina (Eddy and Clarke 2008)

Character 11. Coding for *Acrocanthosaurus* altered to reflect description in text of Porfiri et al 2014 p. 37. Also, coding for *Proceratosaurus* changed from 1 to 0 as no foramen is visible in specimen photos of the holotype taken by PJM. Furthermore, Rauhut et al. (2009) state that, “a true subnarial depression is found only in a small area below the anteroventral end of the nares, where the subnarial borders of the premaxillae are flexed medially” which is not consistent with presence of a deep fossa.

Character 33. Coding changed to State 1 in *Proceratosaurus* to reflect that the base of the nasal horn above the naris is appears fused, and Rauhut et al. (2009) note that an internal suture is not evident either to the naked eye or in CT slices.

Character 67. Numerous non-avian and avian theropod taxa exhibit a pneumatic quadrate. The principal site of quadrate pneumatization in tyrannosaurids appears to be rostral at the juncture of the pterygoid wing and the quadrate shaft above the condyles, which is how the derived state for this character was defined here. Numerous other theropods exhibit a posterior pneumatic opening on the caudal face of the quadrate shaft, and a great degree of variability was described by Tahara and Larsson (2011), who cautioned that topological differences and great variability in quadrate pneumaticity preclude any assumptions about the homologies of which cranial system is involved in pneumatizing the quadrate in non-avian theropods. Therefore we have re-scored all the taxa with a posterior pneumatic opening (*Acrocanthosaurus*, *Sinraptor*, *Sinornithomimus*, *Proceratosaurus*, etc.) as state 0 and added a separate character for a posterior quadrate shaft pneumatic foramen (Character 285).

Character 69. *Sinornithomimus*  is scored as pneumatic (State 1) based on published CT scan data.

Character 80. Fig. 6 D of Porfiri et al (2014) clearly shows that the frontal midline is not well preserved so the width of the element is in some doubt. However, the supratemporal ridge on the frontal appears to diverge caudally where it would be expected to meet the parietal if a parasagittal ridge were present as in tyrannosaurids, and this curvature strongly indicates such a structure is absent.

Character 97: Changed from 1 (slightly opisthocoelous) to 2 (amphyplatyan/ amphicoelous) in *Eotyrannus* based on an isolated cervical centrum belonging to the holotype (PJM personal observation). In coding this, the presence of a convex rostral intercentral articulation is the critical anatomical feature as many theropods have slightly concave posterior intercentral faces, but lack the matching convexity that defines a ball-and-socket joint.

Character 102: None of the known specimens of *Acrocanthosaurus* are sufficiently well preserved to score this character. Furthermore, the cervical vertebrae of *Albertosaurus* do not have accessory articulations (RTMP 68.3.1).

Character 114. Azuma and Currie (2000) describe a single distal caudal of *Fukuiraptor* from the Kitadani Quarry, as having broken zygapohyses so this scoring altered to '?' to reflect the lack of knowledge.

Character 157: The pubis in *Allosaurus*, advanced tyrannosaurids, and *Aerosteon* cannot be considered homologous to either the vertical or retroverted state observed in e.g. *Velociraptor*. The angle between the shaft and the acetabular part of the pubis in these taxa is much closer to what is observed in unambiguously propubic taxa such as *Sinraptor*.

Character 162: Changed from 0 to 1 in *Guanlong* based on photo in supplementary information accompanying the Xu et al 2004 *Guanlong* paper.

Character 176: A large accessory trochanter marks the rostral edge of the anterior trochanter in *Neovenator* (PJM personal observation; Brusatte et al. 2009).

Character 193: Changed from 1 to 2 in *Neovenator* as the figrure in the Brusatte et al.(2009) monograph shows that the fibular fossa is comparable to that of e.g. *Australovenator* both in width and depth. One problem with this character is that it is unclear where fibular width is being measured. I have here assumed it is near the ventral end of the fibular fossa.

Character 212: The foot of *Guanlong* is described as having subequal exposure of the main metatarsals, but is only illustrated in plantar view, so cannot be scored for this trait.

Character 217. This character was introduced by Porfiri et al. (2014), but is miscoded for a number of taxa including *Monolophosaurus*, *Velociraptor*, *Eotyrannus*, *Albertosaurus*, *Compsognathus*, and *Megaraptor. Monolophosaurus*, *Eotyrannus*, and *Megaraptor* are too poorly preserved to determine whether state 1 or 2 is preserved, and the others are recoded based on personal observations/ photographs by PJM.

Character 221: According to Coria & Currie (2000) the proximal tibia is not sufficiently preserved in *Mapusaurus* to record this trait.

Character 226. At least some openings in the ilium of *Mapusaurus* are regarded as pneumatic according to Bell and Coria (2013). Although they interpet some openings as being pathologies, they acknowledge some may connect to interior pneumatic spaces.

Character 234: Altered in *Pitnitzkysaurus as* the depression is clearly present in the MACN 895 (PJM personal observation) and was also specifically mentioend as present in *Pitnitzkysaurus* by Benson & Xu (2008). Coded as present in *Australovenator* based on White et al. (2012) PLoS One. The depression is clearly present in *Albertosaurus* and *Tyrannosaurus* FMNH PR 2081.

Character 245. There are interior spaces in the ilium of *Neovenator*, but no obvious connection to exterior as in e.g. *Aerosteon*. The ilium of *Australovenator* is also clearly

pneumatic, but too poorly preserved to determine if lateral openings are there.

Original character 267 removed as it overlaps with character 80 with both referring to presence of parietal saggital crests as the derived state.

Character 270(Porfiri et al char #272). Fig 7D of Porfiri et al. (2014) shows that the ventral edges of the lateral walls of the basisphenoid recess were not mediolaterally expanded or rugose as in tyrannosaurids (which is what the character was based on), so this is altered from 1 to 0.

Character 271. Having a first alveolus that is smaller than the second is a common trait among most theropods. The distinguishing trait is whether it is substantially smaller than teeth/ alveoli in the middle of the toothrow (Hendrickx and Mateus, 2014), and we re-scored *Australovenator* to follow those authors. The dentary is incomplete in *Fukuiraptor* (Azuma and Currie, 2000) precluding scoring of this trait, an it was changed to “?”.

Character 272. The denticles of *Tyrannosaurus* are block-shaped and perpendicular to the crown axis as in other tyrannosaurids (Brochu, 2003)

References not cited in main paper:

Bell PR, Coria RA. Palaeopathological Survey of a Population of Mapusaurus (Theropoda: Carcharodontosauridae) from the Late Cretaceous Huincul Formation, Argentina. 2013. PLoS ONE; 8: e63409. doi:10.1371/journal.pone.0063409

Benson RBJ, Xu X. The anatomy and systematic position of the theropod dinosaur Chilantaisaurus tashuikouensis Hu, 1964 from the Early Cretaceous of Alanshan, People’s Republic of China. 2008. Geol Mag 145:778–789. doi:10.1017/S0016756808005475

Brochu CA. 2003. Osteology of *Tyrannosaurus rex*: insights from a nearly complete skeleton and high-resolution computed tomographic analysis of the skull. Society of Vertebrate Paleontology Memoir 7:1-138

Eddy DR, Clarke JA. New Information on the Cranial Anatomy of Acrocanthosaurus atokensis and Its Implications for the Phylogeny of Allosauroidea (Dinosauria: Theropoda). 2011. PLoS ONE; 6(3): e17932. doi:10.1371/journal.pone.0017932

Hendrickx C., Mateus O. Abelisauridae (Dinosauria: Theropoda) from the Late Jurassic of Portugal and dentition-based phylogeny as a contribution for the identification of isolated theropod teeth. 2014. Zootaxa; 3759: 1-74.

Rauhut OWM, Milner AC, Moore-Fay S. 2010. Cranial osteology and phylogenetic position of the theropod dinosaur *Proceratosaurus bradleyi* (Woodward, 1910) from the middle Jurassic of England. Zoological Journal of the Linnean Society 158(1):155-195

Tahara R, Larsson HCE. 2011. Cranial pneumatic anatomy of *Ornithomimus* *edmontonicus* (Ornithomimidae: Theropoda) Journal of Vertebrate Paleontology 31(1):127-143

Xu X., Clark JM, Forster CM, Norell MA, Erickson GM, et al. A basal tyrannosauroid dinosaur from the Late Jurassic of China. 2006. Nature; 439: 715-718.
