# Supplementary material for: An Unusual New Theropod with a Didactyl Manus from the Upper Cretaceous of Patagonia, Argentina
Source: PLoS One. 2016 Jul 13;11(7):e0157793. doi: 10.1371/journal.pone.0157793 (PMC4943716; doi:10.1371/journal.pone.0157793)
Supplement: S2 Text — List of forelimb characters taken from [7] with two new characters added and analyzed with Principal Coordinates analysis. (DOCX) [file pone.0157793.s013.docx]

S5: Forelimb characters used for PCO analysis. Charcater A-GG are taken directly from the analysis of Carrano et al. (2012; their chars. 229-260), while the last two were added in this work (chars. 352 and 360 in S2.)

1. Humerus, shape of head: elongate (0), globular (1).†
2. Humerus, longitudinal torsion of shaft: absent (0), present(1).†
3. Although torsion has been scored as absent in some basal tetanurans, such as Xuanhanosaurus, it is present and pronounced in all such taxa.
4. Humerus, size of trochanters relative to midshaft diameter:< (0), > 150% (1) > 250% (2).
5. Humerus, development of internal tuberosity: low/rounded (0), hypertrophied (1).
6. Humerus, length of deltopectoral crest relative to total bone length: < 0.4 (0), 0.43–0.49 (1) > 0.52 (2). An additional state (2) describes the derived condition found primarily in megalosaurids and abelisaurids.
7. Humerus, height of deltopectoral crest: low (0), prominent (1).†
8. Humerus, orientation of deltopectoral crest apex: anteriorly (0), anterolaterally (1).
9. Humerus, relative orientation of proximal & distal condyles in anteroposterior view: parallel, humerus straight (0), distal canted (1). State 1 is present in some allosauroids, and gives the humerus a more twisted appearance, leading some authors to describe it as ‘sigmoid.’ However, the humeral shaft remains curved in only a single plane and so cannot be described as such.
10. Humerus, anterior surface of bone adjacent to ulnar condyle: smooth or gently depressed (0), bears well-defined fossa (1).
11. Humerus, shape of distal condyles: rounded (0), flattened (1).†
12. Radius and ulna, development of radial external tuberosity and ulnar internal tuberosity: low, rounded (0), hypertrophied distal ends of radius and ulna broadened (1). Although previously noted as a spinosaurid synapomorphy, the distal epipodials are also expanded in some carcharodontosaurids (Currie & Carpenter 2000).
13. Radius, shaft: straight (0); curves laterally (1).
14. Radius, development of medial biceps tubercle: small or indistinct (0), hypertrophied (1). The medial biceps tubercle is well developed, forming a distinct tuberosity in *Acrocanthosaurus* and *Poekilopleuron*.
15. Ulna, olecranon process: absent (0), present (1).
16. Ulna, morphology of olecranon process: transversely robust (0); transversely compressed and ‘blade-like’ (1).
17. Ulna, crest extending distally along posterior surface from olecranon process: absent (0), present (1).
18. Ulna, hypertrophied medial and lateral processes on proximal end: absent (0), present (1). The derived state describes the morphology in spinosaurids and splits previous characters that also described structures on the distal ends of the radius and ulna (see 239, above).
19. Ulna, length relative to minimum circumference: stout, < 2.3 (0); gracile > 2.6 (1).
20. Carpus, morphology and articulations of distal carpals: separate dc1 and dc2 over separate metacarpals, flattened proximodistally (0), fused dc1 and dc2, dc1 overlaps metacarpals I and II, flattened proximodistally (1), fused dc1 and dc2, dc1 overlaps metacarpals I and II, strongly arched proximodistally (2).
21. Manus, length relative to length of arm + forearm: <(0), ≥ (1).
22. Manus, composition: digit IV and V present (0), digit IV present, digit V absent (1), MC IV present, IV phalanges and digit V absent (2), digits IV and V absent (3).
23. Manual digits, lengths: III longest (0), II longest (1).
24. Metacarpals, transverse width of proximal articular ends relative to minimum transverse shaft width: < (0), ≥ 2x (1). The proximal ends of the metacarpals are highly expanded in derived carcharodontosaurids.
25. Metacarpal I, length to minimum width ratio: 1.4–1.9 (0), ≥ 2.4 (1).
26. Metacarpal I, length relative to length of metacarpal II: 50% (0), < 50% (1).
27. Metacarpal I, extent of contact with metacarpal II relative to shaft length: < 1/3 (0), 1/2 (1).
28. Metacarpal I, angle between facet for metacarpal II and proximal articular facet: perpendicular (0), obtuse (1).
29. Metacarpal III, position of base relative to those of other metacarpals: at same level (0), on palmar surface (1).
30. Metacarpal III, shape of proximal end: rectangular (0), triangular (1).
31. Metacarpal III, width relative to width of metacarpal II: 50% (0), < 50% (1).
32. Manual ungual I, length:height ratio: < 2.5x (0), > 2.5x (1). This character describes the relative length, and thus size, of manual ungual I, replacing previous characters that describe the size relative to the ulna or radius (and are difficult to score when these elements are not preserved). It is independent of the proportional width of the manual unguals (260, below).
33. Manual unguals, proximal height:width ratio: transversely broad, < 2.0 (0), transversely narrow, > 2.4 (1). The derived state distinguishes the transversely narrow ungual phalanges of some neovenatorids and coelurosaurs from the stout, robust morphology of more basal theropods.
34. Deltopectoral crest, anterior edge: straight or gently arced (0), or lobate (1).
35. 360) Humeral shaft, lateral furrow on proximal half: absent (0) or present (1)
